# *Arabidopsis* AGDP1 links H3K9me2 to DNA methylation in heterochromatin

**DOI:** 10.1038/s41467-018-06965-w

**Published:** 2018-10-31

**Authors:** Cuijun Zhang, Xuan Du, Kai Tang, Zhenlin Yang, Li Pan, Peipei Zhu, Jinyan Luo, Yuwei Jiang, Hui Zhang, Huafang Wan, Xingang Wang, Fengkai Wu, W. Andy Tao, Xin-Jian He, Heng Zhang, Ray A. Bressan, Jiamu Du, Jian-Kang Zhu

**Affiliations:** 1 National Key Laboratory of Plant Molecular Genetics, CAS Center for Excellence in Molecular Plant Sciences, Shanghai Center for Plant Stress Biology, Shanghai Institutes for Biological Sciences, Chinese Academy of Sciences, 201602 Shanghai, China; 2 Department of Horticulture and Landscape Architecture, Purdue University, West Lafayette, IN 47907 USA; 3 University of Chinese Academy of Sciences, 100049 Beijing, China; 4 Department of Biochemistry, Purdue University, West Lafayette, IN 47907 USA; 5 National Institute of Biological Sciences, 102206 Beijing, China; 6 College of Agronomy and Biotechnology, Southwest University, 400715 Chongqing, China; 7 Maize Research Institute, Sichuan Agricultural University, 611130 Chengdu, China

## Abstract

Heterochromatin is a tightly packed form of chromatin that is associated with DNA methylation and histone 3 lysine 9 methylation (H3K9me). Here, we identify an H3K9me2-binding protein, Agenet domain (AGD)-containing p1 (AGDP1), in *Arabidopsis thaliana*. Here we find that AGDP1 can specifically recognize the H3K9me2 mark by its three pairs of tandem AGDs. We determine the crystal structure of the Agenet domain 1 and 2 cassette (AGD12) of *Raphanus sativus* AGDP1 in complex with an H3K9me2 peptide. In the complex, the histone peptide adopts a unique helical conformation. AGD12 specifically recognizes the H3K4me0 and H3K9me2 marks by hydrogen bonding and hydrophobic interactions. In addition, we find that AGDP1 is required for transcriptional silencing, non-CG DNA methylation, and H3K9 dimethylation at some loci. ChIP-seq data show that AGDP1 preferentially occupies long transposons and is associated with heterochromatin marks. Our findings suggest that, as a heterochromatin-binding protein, AGDP1 links H3K9me2 to DNA methylation in heterochromatin regions.

## Abstract (web summary)

DNA methylation and H3K9 dimethylation are two linked epigenetic marks of silenced chromatin in plants that depend on the activity of CMT3/2 and SUVH4/5/6. Here the authors identify AGDP1 as an H3K9me2-binding protein required for heterochromatic non-CG DNA methylation, H3K9 dimethylation, and transcriptional silencing.

## Introduction

In eukaryotic cells, DNA wraps around eight histone protein cores to form nucleosomes, which are further compacted into higher order structures known as chromatin. The chromatin can be divided into euchromatin, where DNA is accessible for transcription, and heterochromatin, where DNA is less accessible for transcription^1^. Modifications on the N-terminal tails of histones, which includes methylation, acetylation, phosphorylation, ubiquitylation, sumoylation, and ADP-ribosylation^2^, provide a mechanism for fine regulation of chromatin states and gene expression.

In plants, H3K9me2 is enriched at constitutively silenced heterochromatin and is required for the silencing of transposable elements (TEs) and other repetitive DNA^3–6^. Besides H3K9me2, DNA methylation is another gene-silencing epigenetic mark. In plants, DNA methylation occurs at all three sequence contexts: CG, CHG (H denotes A, T, or C), and CHH^7–9^. The non-CG methylation (CHG and CHH) is mainly distributed in heterochromatin region and is highly associated with H3K9me2^6^. In *Arabidopsis*, the H3K9me2 mark is solely established by the H3K9 methyltransferases SUVH4 (or KRYPTONITE), SUVH5, and SUVH6, which can recognize methylated DNA^5,10–14^. In the *suvh4/5/6* triple mutant, H3K9me2 levels are strongly reduced globally, and both CHG and CHH methylation are also reduced^5,14,15^. Heterochromatic CHG and CHH methylation, in contrast, are created by the DNA methyltransferases CMT3 and CMT2, respectively, both of which can specifically read the histone H3K9me2 mark^5,16^. Therefore, the heterochromatic non-CG methylation and H3K9me2 are linked together by a cyclic reinforce loop between the writers of non-CG DNA methylation and H3K9me2^6,17^. Whether there is any additional internal linkage between heterochromatic DNA methylation and H3K9me2, however, remains unclear.

Histone modifications can be “read” by specific “reader” modules to translate the upstream histone mark signal to downstream effectors^18^. In plants, the histone mark readers always have some plant-specific features^19^. The “Royal Family” histone readers include several well-known reader domains, such as tudor, Pro-Trp-Trp-Pro (PWWP), chromo, malignant brain tumor (MBT), and agenet (AGD) domains^20^. Among them, the tudor domain has been extensively studied as a histone methylation reader that can recognize multiple histone methylation marks such as H3K4me3, H3K9me3, H3K36me, and H4K20me2/3, as well as some histone arginine methylation marks^21^. AGD was initially defined as a type of plant-specific tudor domain but also exists in some animal proteins such as human Fragile X Mental Retardation Protein (FMRP)^20^. The tandem AGD of FMRP has been reported to bind to the H3K79me2 mark both in vivo and in vitro^22,23^. Although some plausible aromatic residues for the methyllysine binding have been implicated in the structure of FMRP, the molecular mechanism of the recognition of methylated histone by AGD is still unknown, due to a lack of any histone peptide complex structure^24^.

Here, we identify a previously uncharacterized H3K9me2-binding protein, which was named AGD-containing protein 1 (AGDP1). The AGDP1 protein possesses three pairs of tandem AGDs (AGD12, AGD34, and AGD56), all of which can specifically recognize the H3K9me2 mark. Our biochemical and structural studies reveal the molecular basis for the specific recognition of the dual marks H3K4me0 and H3K9me2 by the three tandem AGD cassettes of AGDP1. We also find that AGDP1 is required for transcriptional silencing, CHG and CHH methylation, and H3K9 dimethylation at some loci. Genome-wide mapping of AGDP1-binding sites by chromatin immunoprecipitation (ChIP)-seq show that most AGDP1-binding sites are in TE regions and are associated with the heterochromatin mark H3K9me2. The enrichment of multiple H3K9me2-binding sites on a single AGDP1 protein provides a structural basis for the genome-wide localization of AGDP1 to the H3K9me2-enriched heterochromatin. Our studies identify an H3K9me2-binding protein that regulates heterochromatic DNA methylation and reveal the molecular basis for the specific recognition of H3K9me2 mark, thus establishing an additional linkage between H3K9me2 and heterochromatic DNA methylation.

## Results

### Identification of AGDP1 as an H3K9me2-binding protein

**Fig. 1:**
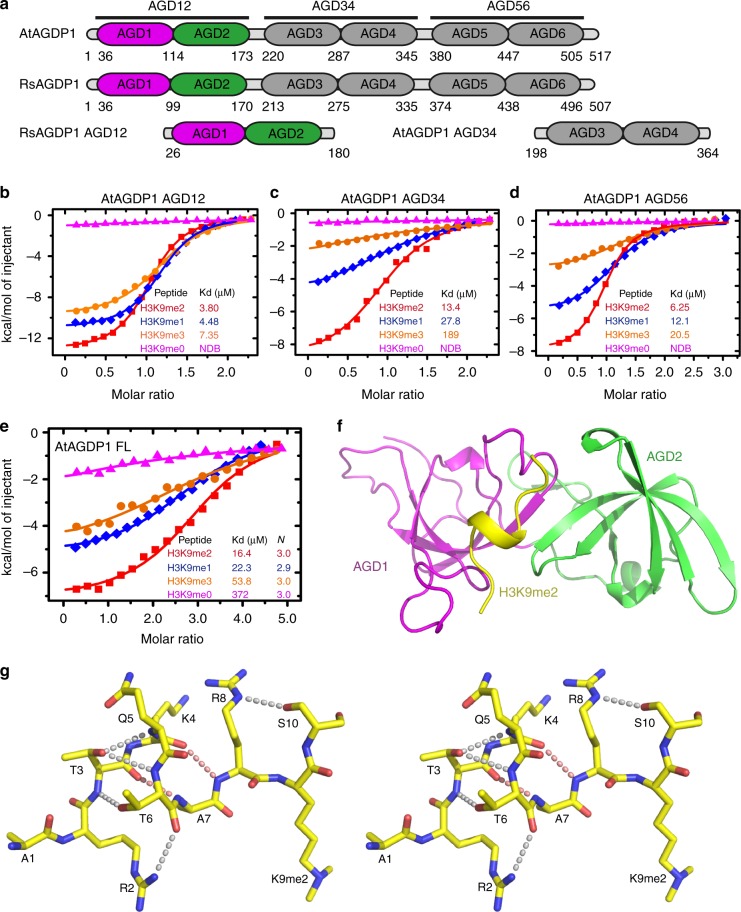
Structure of the RsAGDP1 AGD12–H3K9me2 complex. **a** A schematic representation of the domain architecture of AtAGDP1 (upper panel), RsAGDP1 (middle panel), and the constructs of RsAGDP1 AGD12 and AtAGDP1 AGD34 used for crystallization in this research (lower panel). **b**–**d** ITC-binding curves between different methylated H3K9 peptides and AGD12 (**b**), AGD34 (**c**), and AGD56 (**d**) of AtAGDP1 show that all three tandem AGD cassettes recognize methylated H3K9 marks with a preference on H3K9me2. NDB, no detectable binding. **e** ITC-binding curves between different methylated H3K9 peptides and full-length AtAGDP1 show that the full-length AGDP1 recognize methylated H3K9 with a preference on H3K9me2 and the bindings have a binding ratio near 3. FL, full-length. **f** The overall structure of the RsAGDP1 AGD12-H3K9me2 complex is shown in ribbon diagram with the AGD1, AGD2, and H3K9me2 peptide colored in magenta, green, and yellow, respectively. The peptide adopts a unique helical conformation. **g** A stereo view of the intra-molecular hydrogen-bonding interactions within the H3K9me2 peptide. The main chain–main chain hydrogen bonds and side chain–main chain hydrogen bonds are highlighted by dashed red and silver lines, respectively

To identify readers of H3K9 methylation marks, we incubated the nuclear extracts of *Arabidopsis* flowers with synthesized biotinylated H3(1-21)K9me1, H3(1-21)K9me2, H3(1-21)K9me3, or H3(1-21) peptides. The peptides and their captured proteins were affinity purified with streptavidin beads and were analyzed by mass spectrometry. Consistent with previous reports, we found that CMT3, CMT2, and ENHANCED DOWNY MILDEW 2 (EDM2) were present in the H3K9me1, H3K9me2, and H3K9me3 pull-down products but not in H3 pull-down products (Supplementary Data 1)^5,16,25^. CHROMATIN REMODELING FACTOR 17 (CHR17) was present in all of the peptide pull-down products, indicating that CHR17 can bind to histone H3 tails with or without H3K9me modifications. Interestingly, an AGD-containing protein was identified from the H3K9me1, H3K9me2, and H3K9me3 pull-down samples but not from the H3 pull-down samples. This protein was subsequently named AGDP1 (Supplementary Data 1). AGDP1 possesses six AGDs that can be divided into three tandem AGD cassettes: AGD12, AGD34, and AGD56 (Fig. 1a). To map the histone-binding region on AGDP1, we performed isothermal titration calorimetry (ITC)-based binding assay. Intriguingly, all three tandem AGD cassettes (AGD12, AGD34, and AGD56) can specifically bind to the methylated H3K9 mark with preference on H3K9me2 (Figs. 1b–d), suggesting that a single AGDP1 protein has multiple H3K9me2-binding sites. We further measured the binding between the full-length AtAGDP1 and different H3K9 methylated peptides. Consistently, the full-length AtAGDP1 also showed a preference toward H3K9me2. This binding yields a binding stoichiometry of about 3 (Fig. 1e), confirming that a single AGDP1 protein can bind to three H3K9me2 tails.

### Structure of AGD12 in complex with an H3K9me2 peptide

To investigate the molecular mechanism of the recognition of H3K9me2 by AGDP1, we performed structural studies. The AtAGDP1 AGD12 only produced low-resolution crystals that were not suitable for structure determination. As an alternative, we successfully obtained high-quality crystals of AGD12 of the *Raphanus sativus* AGDP1 (RsAGDP1), which displays 83% sequence identity to AtAGDP1 and which also specifically binds to the H3K9me2 mark (Fig. 1a and Supplementary Fig. 1a–c), in complex with an H3(1-15)K9me2 peptide. The structure was determined at 1.9 Å resolution (Fig. 1f, Supplementary Fig. 1d, and Supplementary Table 1). The AGD12 of RsAGDP1 adopts a classical tandem tudor-like conformation (Fig. 1f). The two AGDs (AGD1 and AGD2) display the canonical AGD/tudor family twisted β-barrel-like fold (Fig. 1f). The overall RsAGDP1 AGD12 structure resembles the reported structure of the tandem AGD of FMRP with a superimposition RMSD of 1.9 Å (Supplementary Fig. 1e)^24^.

### The unique helical conformation of the H3 peptide

Interestingly, the H3K9me2 peptide adopts a unique helical conformation with the four residues from H3K4 to H3A7 forming a short α-helix (Fig. 1f and Supplementary Fig. 1d). In addition to the α-helix featured main chain–main chain hydrogen bonds, the side chains of H3R2, H3T3, H3T6, and H3R8 also form extensive intra-molecular hydrogen-bonding interactions to stabilize the unique conformation of the peptide (Fig. 1g). The intra-molecular hydrogen bonds constrain the peptide into a contractive conformation that is distinct from the commonly observed β-strand-like or extended loop-like conformations^18^. This type of H3 tail conformation has been observed in the structure of Bromodomain Adjacent to Zinc Finger 2A (BAZ2A) PHD finger in complex with an H3(1-10) peptide with the superimposition of the two peptide yielding an RMSD of 0.6 Å (Supplementary Fig. 1f)^26^, suggesting a possible common feature that enables the H3 N-terminus to adopt a helical conformation in binding to certain readers. We think that the unique helical conformation of the peptide may reduce the distance between its N- and C-termini and thus enable multiple interactions of the peptide by the reader over a short distance.

### Molecular interactions between H3K9me2 and AGD12

**Fig. 2:**
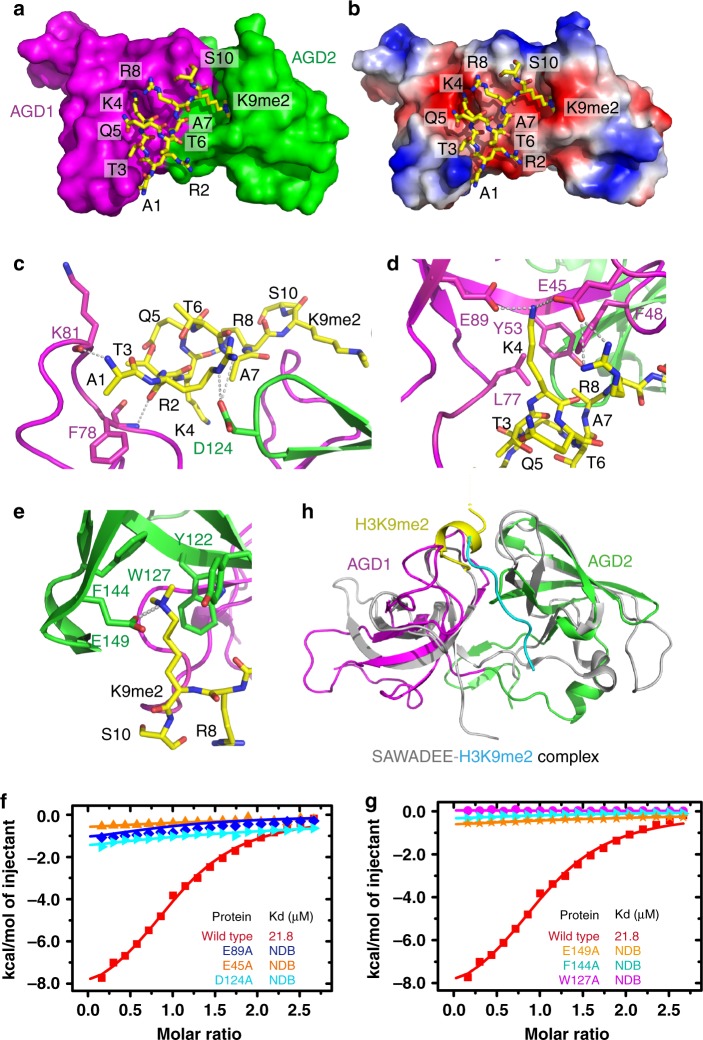
Interactions between the H3K9me2 peptide and RsAGDP1 AGD12. **a** A surface view of the RsAGDP1 AGD12 with AGD1 and AGD2 colored in magenta and green, respectively. The H3K9me2 peptide is shown in stick representation. The side chains of H3K4me0 and H3K9me2 insert into two adjacent surface pockets on AGD1 and AGD2, respectively. **b** An electrostatics surface view of the RsAGDP1 AGD12 with the H3K9me2 peptide in stick representation. The peptide binds at a large negatively charged surface with H3K4me0 and H3K9me2 inserting into two negatively charged surface pockets, respectively. **c**-**e** The detailed recognition of H3A1 and H3R2 (**c**), H3K4, H3A7, and H3R8 (**d**), and H3K9me2 (**e**). The interacting residues and the hydrogen bonds are highlighted in stick and dashed silver lines, respectively. **f**-**g** The ITC-binding curves between H3K9me2 and RsAGDP1 AGD12 mutations disrupting the H3R2, H3K4, and H3R8 (**f**) or the H3K9me2 (**g**) binding residues show that the mutations of critical residues significantly impair the binding between AGD12 and H3K9me2. **h** A superimposition of RsAGDP1 AGD12-H3K9me2 complex (in color) and SHH1 SAWADEE-H3K9me2 complex (in silver, PDB code: 4IUT). The H3K9me2 peptide from the SHH1 SAWADEE complex is colored in cyan. The AGDP1 AGD12 and SHH1 SAWADEE generally adopt a similar folding topology with their binding H3K9me2 peptides locating at the shorter and longer edge of the two AGD/tudor domain interface, respectively

The AGD12 of RsAGDP1 employs a large negatively charged surface to accommodate the H3 peptide with the side chains of H3K4 and H3K9me2 inserting into two adjacent surface pockets of the AGD1 and AGD2, respectively (Figs. 2a, b), highlighting the important role of H3K4 and H3K9me2 in the recognition. Other peptide residues also contribute to the interaction with the AGD12 of AGDP1. In detail, the H3A1 and H3R2 form main chain–main chain hydrogen bonds with the Lys81 and Phe78 of AGD1, respectively (Fig. 2c). The guanidino group of H3R2 forms hydrogen-bonding and salt bridge interactions with the Asp124 of AGD2 (Fig. 2c). The unmodified H3K4 forms hydrogen-bonding and salt bridge interactions with the acidic residues Glu89 and Glu45 from the AGD1, and Glu45 also interacts with the unmodified H3R8 (Fig. 2d). The side chain methyl group of H3A7 is accommodated by a shallow hydrophobic pocket formed by Phe48, Tyr53, and Leu77 via hydrophobic interactions (Fig. 2d). The dimethyllysine of H3K9me2 is accommodated by a classical aromatic cage formed by Tyr122, Trp127, and Phe144 of AGD2 by hydrophobic and cation-π interactions like most reported methyllysine readers (Fig. 2e)^18^. In addition to the aromatic cage, a Glu149 residue can form a hydrogen bond with the dimethylammonium proton to strengthen the binding and facilitate the selectivity for dimethyllysine (Fig. 2e). The H3T3, H3Q5, H3T6, and H3S10 do not have specific interactions with the protein. We further performed structure-based mutagenesis studies. Except Y122A, which precipitated heavily, the mutations that disrupted the H3R2-, H3K4me0-, H3R8-, or H3K9me2-binding pocket significantly impaired the binding between AGD12 and the H3K9me2 peptide (Figs. 2f, g). We further investigated the influence of H3K4 methylation to the recognition by ITC. The H3K4me1K9me2 and H3K4me2K9me2 peptides show slightly decrease to the binding by AGD12 (Supplementary Fig. 1g), indicating that the tandem AGD of AGDP1 can biochemically tolerate the H3K4me1 and H3K4me2. The H3K4me3K9me2 peptide displays a nearly fivefold decrease of the binding (Supplementary Fig. 1g), indicating that the higher H3K4 methylation state leads to a decreased binding by AGD12. Therefore, we conclude that AGD12 prefers an H3K4me0K9me2 state of the histone tail.

The peptide-binding interface of AGD12 is different from that of the SHH1 SAWADEE domain, indicating a different histone-binding interface on the tandem tudor-like domains (Fig. 2h). Note that all residues involved in the peptide recognition are strictly conserved among multiple plant species, including *Arabidopsis thaliana*, which was used for our functional analyses in this study (Supplementary Fig. 2).

### Mechanism of the H3K9me2 recognition by AGD34 and AGD56

**Fig. 3:**
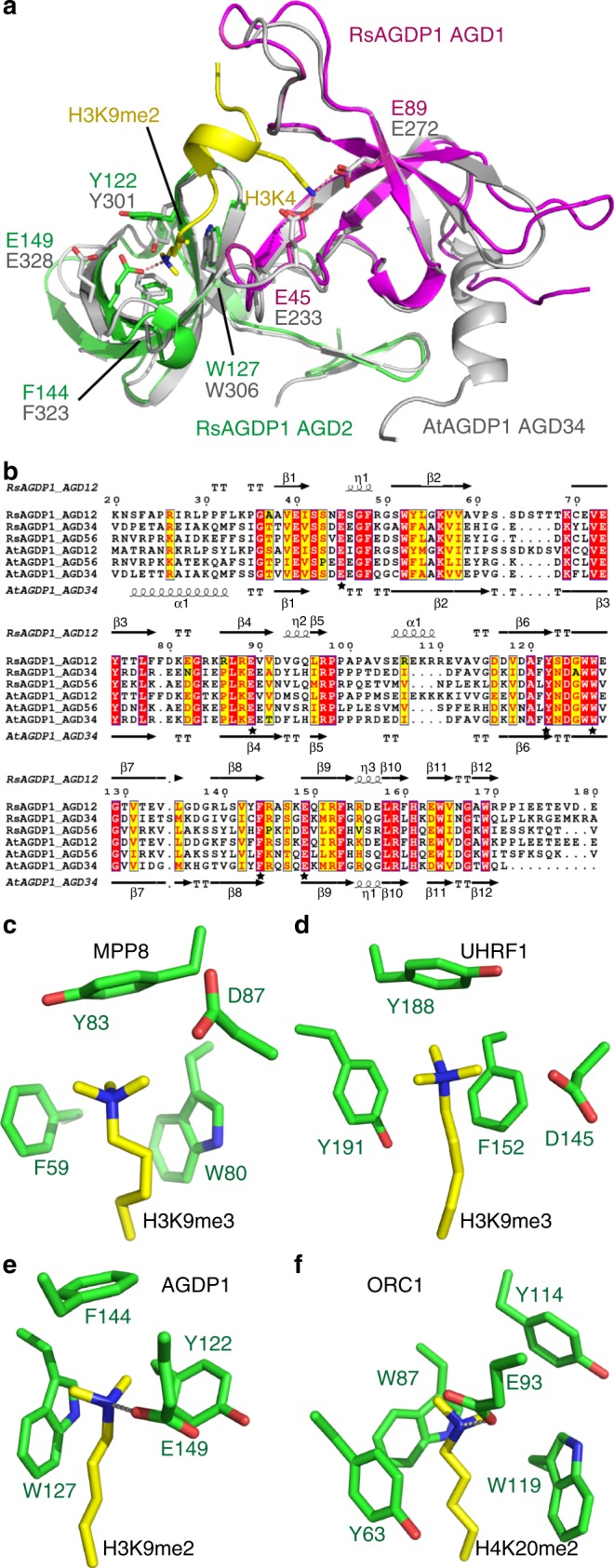
AGD12, AGD34, and AGD56 of AGDP1 recognize methylated H3K9 by the same mechanism. **a** A superimposition of the structures of RsAGDP1 AGD12-H3K9me2 complex (in color) and AtAGDP1 AGD34 (in silver) indicate that the AGD12 and AGD34 adopt almost identical overall structures. The H3K4- and H3K9me2-binding residues are highlighted in sticks and they are conserved and occupy the same positions. **b** A structure-based sequence alignment of AtAGDP1 AGD12, AGD34, and AGD56, and RsAGDP1 AGD12, AGD34, and AGD56 with the secondary structures of RsAGDP1 AGD12 and AtAGDP1 AGD34 highlighted on the top and bottom of the alignment, respectively. The H3K4- and H3K9me2-binding residues are highlighted by stars, and most of them are conserved. We therefore conclude that all the tandem AGD cassettes of AtAGDP1 and RsAGDP1 recognize H3K9me2 via a shared mechanism with conserved peptide-interacting residues. **c**-**f** The comparison of the methyllysine-aromatic cage interaction of MPP8 (**c**, PDB code: 3R93), UHRF1 (**d**, PDB code: 4GY5), AGDP1 (**e**), and ORC1 (**f**, PDB code: 4DOW). The residues are shown in stick representation with the reader and methyllysine residues colored in green and yellow, respectively. The hydrogen bonds between dimethyllysine and the negatively charged residues are highlighted in dashed silver lines

We also performed structural studies on AGD34 and AGD56 of AtAGDP1. We obtained the free-form structure of AtAGDP1 AGD34 (Fig. 3a and Supplementary Table 1). The overall structure of AtAGDP1 AGD34 resembles that of RsAGDP1 AGD12 with all the peptide-binding residues, especially the H3K4me0- and H3K9me2-binding residues, conserved and occupying the same positions (Fig. 3a). The structure-based sequence alignment of AtAGDP1 and RsAGDP1 tandem AGD cassettes indicates that most of the peptide-interacting residues, especially the H3K4- and H3K9me2-binding residues, are conserved (Fig. 3b). The RsAGDP1 Phe78 and Lys81 are not conserved. However, both Phe78 and Lys81 contribute to the interactions with AGDP1 by their main chains. Considering that our ITC data indicated that all three tandem AGD cassette have the same H3K9me2-binding preference, we conclude that the AGD12, AGD34, and AGD56 cassettes of AGDP1 share the same H3K9me2 recognition mechanism.

### Structural basis for the preference of dimethyllysine

The tandem AGDs of AGDP1 bind to different methylated states of H3K9 with the strongest binding to dimethyllysine, lower binding to monomethyllysine and trimethyllysine, indicating a preference for dimethylation. In contrast, most of the reported methyllysine readers use the same aromatic cage to recognize the methyllysine with a preference for higher methylation states of trimethyllysine. To elucidate the molecular basis for this preference, we compared their aromatic cages. We used the H3K9me3 preferred binding protein M-phase phosphoprotein 8 (MPP8) and Ubiquitin-like, Containing PHD and RING Finger Domains, 1 (UHRF1) as references^27–30^. MPP8 and UHRF1 have methyllysine-binding aromatic cages possessing relative open conformations (Figs. 3c, d). Whereas one side of the trimethyllysines is embedded in the aromatic cages, the other sides of the trimethyllysines are exposed (Figs. 3c, d). Although there are also negatively charged residues near the aromatic cages, they are aligned aside of the cage and leave the pocket open to accommodate bigger head group of the trimethyllysine (Figs. 3c, d)^27–30^. Therefore, we think that these negatively charged residues in MPP8 and UHRF1 mainly function in neutralizing the charge of the methyllysines. In contrast, RsAGDP1 AGD12 has a different type of aromatic cage that is similar to the BAH domain of mouse Origin of Replication Complex Subunit 1 (ORC1) (Figs. 3e, f)^31^. The aromatic residues are similar to MPP8 or UHRF1, but the negatively charged residues are located differently (Figs. 3c–f). In AGDP1 and ORC1, the aromatic cages are covered by the negatively charged residues, forming closed cages to accommodate the methyllysine (Figs. 3e, f)^31^. The negatively charged residues not only neutralize the charge of the methyllysines but also form direct hydrogen bonds with the free protons of the dimethyllysine, leading to a preference to the free proton containing dimethyllysine but not the trimethyllysine. In addition, the closed pockets of AGDP1 and ORC1 are too narrow to accommodate the trimethyllysine, resulting in the preference for the dimethyllysine and decreased binding to trimethyllysine. Although the monomethyllysine can also form a direct hydrogen bond with and can be accommodated by AGDP1/ORC1 type binding pocket, the single methyl group has an obviously decreased binding by the aromatic cage, resulting a weaker binding than the dimethyllysine. However, there are still minor differences between the tandem AGD cassettes. For example, the binding affinity AtAGD12 to H3K9me2 (3.8 μM) is only 1.2-fold higher than H3K9me1 (4.48 μM) (Fig. 1b). In contrast, the AtAGD34 and AtAGD56 binds to H3K9me2 about twofold higher than H3K9me1 (Figs. 1c, d), although the peptide-binding regions are quite conserved (Fig. 3b). The relative preferences of H3K9me3 and H3K9me1 by different tandem AGD cassettes are different, too. A possible reason could be that some minor differences outside the H3K9me2-binding pocket may slightly change the shape and charge of the H3K9me2-binding pockets, leading to different sub-level preferences. These sub-level preferences may participate in the fine tuning functions of different tandem AGD cassettes and provide a plausible additional layer of regulation.

### AGDP1 contributes to transcriptional silencing at some loci

**Fig. 4:**
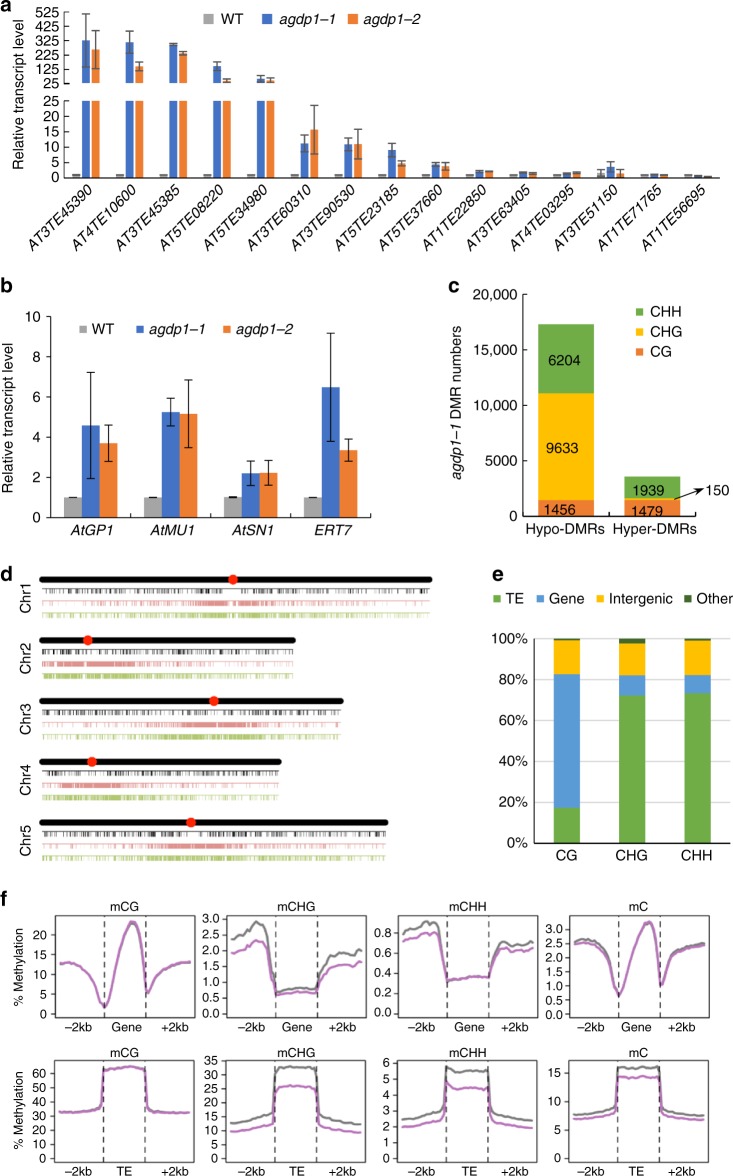
AGDP1 contributes to transcriptional silencing and non-CG methylation at some loci. **a** The transcript levels of the indicated TEs were determined by quantitative RT-PCR in the wild-type, *agdp1-1*, and *agdp1-2*. Error bars are SDs of three biological replicates. **b** The RNA transcripts of *AtMU1*, *AtGP1*, *AtSN1*, and *ERT7* were detected by quantitative RT-PCR in the wild-type, *agdp1-1*, and *agdp1-2*. Error bars are SDs of three biological replicates. **c** The numbers of CG, CHG, and CHH hypo-DMRs and hyper-DMRs identified in *agdp1-1*. **d** The distribution of hypo-DMRs throughout the five chromosomes. Black, pink, and green bars represent CG, CHG, and CHH hypo-DMRs, respectively. Red circles indicate centromeres. **e** Composition of the genomic location of the hypo-DMRs. TE, transposable element. **f** Average DNA methylation levels along protein-coding genes (upper) and TEs (lower) and the 2-kb flanking regions in different cytosine contexts. Gray and pink curves represent DNA methylation levels of wild-type and *agdp1-1*, respectively

To investigate the function of AGDP1, we obtained two individual T-DNA insertion mutants, *agdp1-1* (Salk_134878) and *agdp1-2* (Salk_130936), from ABRC (Supplementary Fig. 3a–c). Under normal growth conditions, we did not observe developmental phenotypes for *agdp1-1* and *agdp1-2* mutants compared with the wild-type. Because AGDP1 can bind to the silencing mark H3K9me2, we wondered whether AGDP1 is required for transcriptional silencing. We performed RNA deep sequencing assay with five replicates for wild-type and *agdp1-1*, and three replicates for *suvh4/5/6*. We found that only 15 TEs and 42 protein-coding genes (*P* < 0.05; log2(fold-change) > 1; Cufflinks) were upregulated in the *agdp1-1* mutants (Supplementary Data 2). The upregulated TEs were validated by quantitative reverse transcription (RT)-PCR analyses, which showed that 12 of them were upregulated in both *agdp1-1* and *agdp1-2* mutants (Fig. 4a). Ninety-one percent (11 of 12) of upregulated TEs and 57% (24 of 42) of upregulated protein-coding genes in *agdp1-1* mutants were derepressed in *suvh4/5/6* mutants (Supplementary Data 2), suggesting that some of the SUVH4/5/6-dependent loci are under regulation by AGDP1. We also determined the expression levels of the previously characterized hypermethylated loci *AtMU1*, *AtGP1*, *AtSN1*, and *ERT7* by quantitative (RT)-PCR and found that these four loci were also upregulated in *agdp1-1* and *agdp1-2* mutants (Fig. 4b). These four loci were not identified as upregulated TEs or genes by RNA-Seq, perhaps due to their relatively low expression levels. In any case, the results suggest that there may be additional loci that are regulated by AGDP1.

### AGDP1 is required for both CHG and CHH methylation

To determine whether AGDP1 has a role in DNA methylation, we performed whole-genome bisulfite sequencing (WGBS) for the wild-type and *agdp1-1* mutant plants. We identified 1456 CG, 9633 CHG, and 6204 CHH hypomethylated differentially methylated regions (hypo-DMRs) in *agdp1-1* compared with the wild-type; however, we identified only 1479 CG, 150 CHG, and 1939 CHH hyper-DMRs in *agdp1-1* (Fig. 4c and Supplementary Data 3). The large number of CHG and CHH hypo-DMRs in *agdp1-1* suggested that AGDP1 is required for both CHG and CHH methylation in the genome. The CHG and CHH hypo-DMRs are mainly located in pericentromeric heterochromatin regions (Fig. 4d). In addition, most of the CHG and CHH hypo-DMRs are in TE regions (Fig. 4e). Through classification analysis for TE families, we found that DNA/En-Spm, LTR/*Copia*, LTR/*Gypsy* are enriched in both CHG and CHH hypo-DMRs (Supplementary Data 4). Previously, Panda et al. divided TEs to truncated TEs and full-length TEs^32^. Compared with the truncated TEs, the full-length TEs are enriched in both CHG and CHH hypo-DMRs of *agdp1-1* (Supplementary Data 4). By analyzing the average DNA methylation levels along protein-coding genes and TEs in different cytosine contexts, we found that CHG and CHH methylation are decreased in TE regions and in the 2-kb flanking regions of protein-coding genes in the *agdp1-1* mutant (Fig. 4f). In contrast, the CG methylation was not changed in protein-coding genes and TE regions in the *agdp1-1* mutant, suggesting that *agdp1* mainly affects CHG and CHH methylation. The reduction of DNA methylation in *agdp1-1* and *agdp1-2* mutants was confirmed by chop-PCR (Supplementary Fig. 3d).

### *agdp1* affects SUVH4/5/6-dependent CHG and CHH methylation

**Fig. 5:**
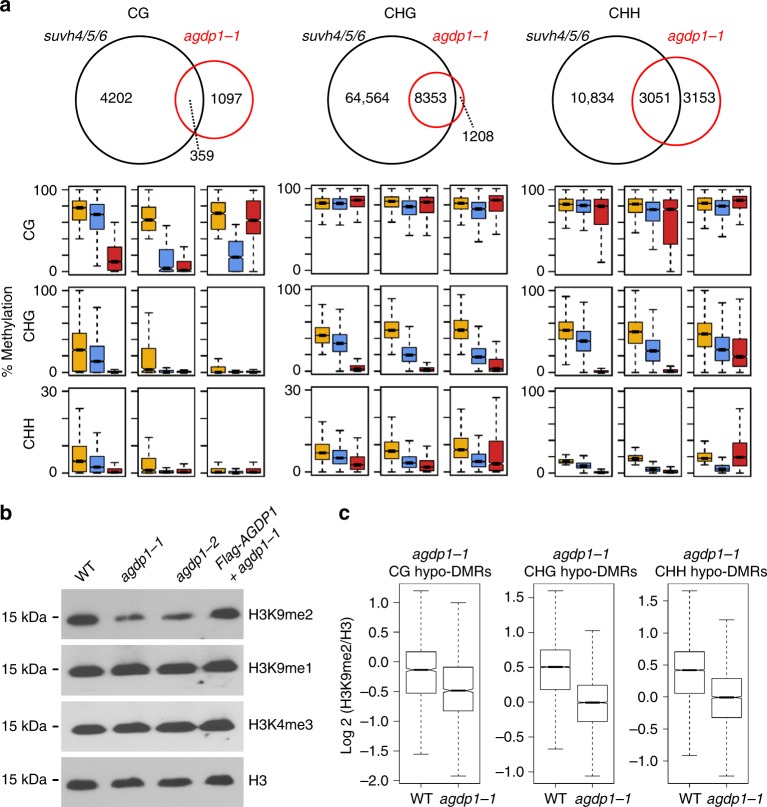
*agdp1* affects H3K9 dimethylation and *suvh4/5/6*-dependent CHG and CHH methylation. **a** Venn diagrams showing the overlap of hypo-DMRs between *agdp1* and *suvh4/5/6* in CG, CHG, and CHH contexts. Box plots represent CG, CHG, and CHH methylation levels of overlapping or unique hypo-DMRs in the upper Venn diagrams. Yellow, blue, and red boxes indicate DNA methylation levels in the wild-type, *agdp1-1*, and *suvh4/5/6*, respectively. **b** Western blot analysis of H3 lysine methylation status in wild-type Col-0 plants and in *agdp1-1*, *agdp1-2*, and *Flag-AGDP1* transgenic plants in the *agdp1-1* mutant background with the antibodies specified on the right. **c** Box plots of levels of H3K9me2 relative to H3 in *agdp1-1* CG, CHG, and CHH hypo-DMRs. The *y* axis represents the ChIP-seq read density normalized by H3

Both CHG and CHH methylation are reduced in the *suvh4/5/6* triple mutant^15^. The finding that AGDP1 helps maintain CHG and CHH methylation levels in the genome led us to compare the hypo-DMRs of *agdp1-1* and *suvh4/5/6* mutants. We found that about 87% of *agdp1-1* CHG-hypomethylated sites overlapped with *suvh4/5/6* CHG-hypomethylated sites (Fig. 5a). For CHH methylation, 49% of *agdp1-1* CHH-hypomethylated sites overlapped with *suvh4/5/6* CHH-hypomethylated sites (Fig. 5a). Box plots analysis showed that the CHG and CHH methylation levels at the *suvh4/5/6*-specific loci also appeared decreased in the *agdp1-1* mutant (Fig. 5a), although the decreases in *agdp1-1* were insufficient to cause the loci to be considered hypo-DMRs. These results suggest that SUVH4/5/6-dependent CHG and CHH methylation are reduced in the *agdp1-1* mutant.

To further analyze the effect of the *agdp1-1* mutation on DNA methylation pathways, we performed hypo-DMR overlap analysis between *agdp1-1* and *cmt2*, *cmt3*, *drm1/2*, or *ddm1* mutants with published WGBS data^15,33^. We found that most of the CHG hypo-DMRs of *agdp1-1* overlapped with *cmt3* and *ddm1* (Supplementary Fig. 4). For the CHH context, 45%, 28%, and 54% of the *agdp1-1* CHH-hypomethylated sites overlapped with *cmt2*, *drm1/2*, and *ddm1*, respectively (Supplementary Fig. 4). Consistent with the overlap analysis, box plot analysis showed that at *agdp1-1* CHG hypo-DMRs, there was a strong loss of CHG methylation in *suvh4/5/6*, *cmt3*, and *ddm1* mutants (Supplementary Fig. 5). At *agdp1-1* CHH hypo-DMRs, the CHH methylation was reduced in *suvh4/5/6*, *cmt2, drm1/2*, and *ddm1* mutants (Supplementary Fig. 5).

To determine whether the *agdp1* mutation affects H3K9 dimethylation levels, we investigated the global level of H3K9me2 in the wild-type and in the *agdp1-1* and *agdp1-2* mutants with immunoblotting assays. The results showed that H3K9me2 levels were reduced in *agdp1-1* and *agdp1-2*, whereas the levels of H3K9me1 and H3K4me3 in *agdp1-1* and *agdp1-2* mutants were comparable to that in the wild-type (Fig. 5b). Moreover, the reduced H3K9me2 level in *agdp1-1* was fully rescued by the *pAGDP1-Flag-AGDP1* transgene (Fig. 5b). To further confirm the reduction of H3K9me2 levels in *agdp1*, we performed H3K9me2 and H3 ChIP-Seq in wild-type and *agdp1-1*. Box plot analysis showed that the H3K9me2 levels in *agdp1-1* mutants are reduced at AGDP1-target sites (Fig. 5c). These results indicate that *agdp1* affects SUVH4/5/6-dependent CHG and CHH methylation and has a global effect on H3K9 dimethylation. Our RNA-seq data showed that the expression levels of *CMT2*, *CMT3*, *SUVH4*, *SUVH5*, *SUVH6*, and *DDM1* were not affected by mutation of *agdp1-1* (Supplementary Data 2). This indicates that the reductions of non-CG methylation and of H3K9me2 in *agdp1* mutants is not caused by the reduced expression of these genes.

### AGDP1 is enriched in heterochromatin

**Fig. 6:**
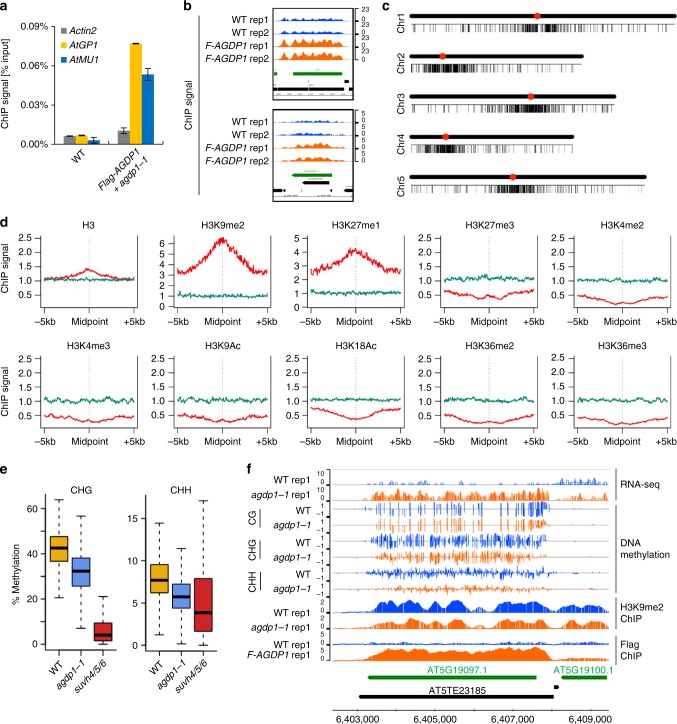
AGDP1 is enriched in heterochromatin. **a** AGDP1 is enriched at *AtGP1* and *AtMU1* as determined by ChIP-qPCR. ChIP signals were quantified relative to input DNA. *Actin2* was used as a negative control locus. Error bars are SDs of three biological replicates. **b** ChIP-seq results show that AGDP1 is enriched at *AtGP1* (upper) and *AtMU1* (lower). Two replicates of ChIP-seq data from *Flag-AGDP1* transgenic plants and from wild-type control plants are shown. The *y* axis is normalized read counts per million. The normalization factor is calculated by the total mapped reads. **c** The distribution of AGDP1-enriched peaks throughout the five chromosomes. Black bars represent AGDP1 peaks. Red circles indicate centromeres. **d** Association of different histone modifications at regions surrounding the AGDP1 peaks. Simulation regions (green) served as control regions. **e** Box plots representing CHG and CHH methylation levels of 1914 AGDP1-binding regions. **f** RNA expression, DNA methylation, H3K9me2 levels, and AGDP1 enrichment at a representative region. The *y* axis is normalized read counts per million (ChIP-seq) or ten million (RNA-seq). The normalization factor is calculated by the total mapped reads

Subnuclear localization analyses showed that, the fluorescence of the control green fluorescent protein (GFP), was dispersed in the nuclear and excluded from the nucleolus regions, GFP-AGDP1 fluorescence showed similar localization patterns with 4,6-diamidino-2-phenylindole (DAPI) staining, except for the strong fluorescence signal within the nucleolus (Supplementary Fig. 6). To determine whether AGDP1 directly associates with heterochromatin, we performed ChIP assays with *pAGDP1-Flag-AGDP1* transgenic plants. The transgene was functional in vivo, because its expression fully rescued the phenotypes of reduced H3K9me2 level and increased expression of *AtGP1* and *AtMU1* in the *agdp1-1* mutant (Fig. 5b and Supplementary Fig. 7). ChIP-qPCR results showed that AGDP1 is enriched at *AtGP1* and *AtMU1* loci but not at the negative control locus *Actin2* (Fig. 6a). To further map the genome-wide occupancy of AGDP1, we carried out ChIP-seq analysis (ChIP followed by sequencing) for Flag-AGDP1 and wild-type control samples. The mapped reads at *AtGP1* and *AtMU1* loci showed that AGDP1 is enriched at these two TEs (Fig. 6b). The genome-wide analysis revealed a total of 1914 AGDP1 enrichment peaks (Supplementary Data 5). The distribution analysis throughout the five chromosomes showed that AGDP1 peaks are concentrated in centromeric and pericentromeric regions (Fig. 6c), which is similar to that of *agdp1* CHG and CHH hypo-DMRs (Fig. 4d). To determine which histone marks are associated with AGDP1-binding peaks, we compared the AGDP1 occupancy profiles with published genome-wide histone modification data^34,35^. Compared with the randomly selected genomic regions with the same length distribution as the AGDP1 peaks, regions encompassing the AGDP1 peaks show a slight increase in the level of H3 (Fig. 6d), indicating a higher nucleosome density in AGDP1 targets. In addition, AGDP1 peaks were found to be positively associated with the heterochromatin marks H3K9me2 and H3K27me1 (Fig. 6d). In contrast, AGDP1 peaks were found to be negatively associated with most active histone marks compared with control regions (Fig. 6d). Consistently, Scatter plot analysis showed that AGDP1 enrichment is highly correlated with H3K9me2 modification (Supplementary Fig. 8a). At AGDP1-binding regions, the CHG and CHH methylation and H3K9me2 levels were decreased in *agdp1-1* (Fig. 6e and Supplementary Fig. 8b). Snapshots of the genome browser showed that AGDP1 is enriched at four representative TE regions and required both for the silencing and the non-CG methylation of these four TEs (Fig. 6f and Supplementary Fig. 9).

### AGDP1 preferentially binds to long TEs

**Fig. 7:**
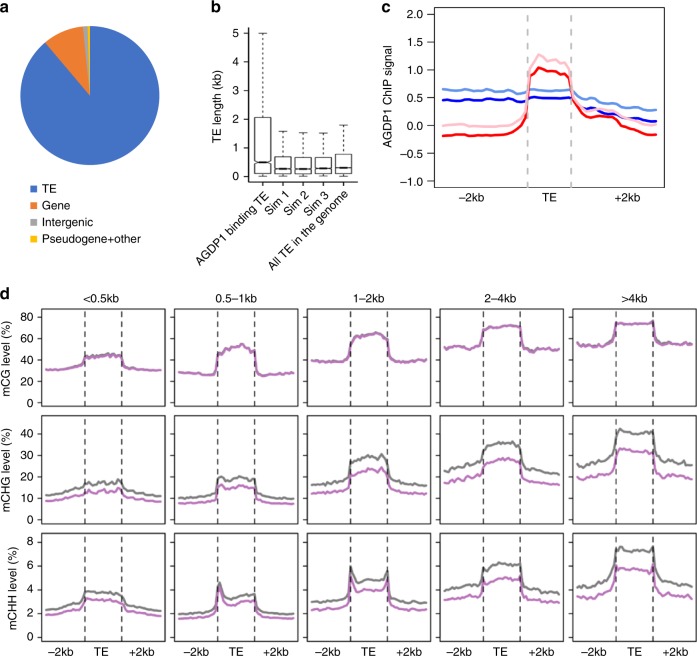
AGDP1 preferentially binds to long TEs. **a** Composition of the genomic locations of the AGDP1-enriched peaks. TE, transposable element. **b** Box plots of the length of TEs that are bound to AGDP1 and of all TEs in the *Arabidopsis* genome. Only TEs with >80% ovelap with ChIP-seq peaks were defined as “AGDP1 bound TEs”. Three sets of randomly distributed peaks with the same length distribution of the AGDP1 peaks served as control regions. **c** Metaplot analysis of AGDP1 enrichment over long and short TEs. Blue and light blue curves represent ChIP signals of replicate 1 and 2 over short TEs (length ≤ 4 kb). Red and light red curves represent ChIP signals of replicate 1 and 2 over long TEs (length > 4 kb), respectively. **d** Average DNA methylation levels along different length of TEs in different cytosine contexts. Gray and pink curves represent DNA methylation levels of wild-type and *agdp1-1*, respectively

Our WGBS results showed that most of the CHG and CHH hypo-DMRs are located in TE regions (Fig. 4e). Similar to the distribution of CHG and CHH hypo-DMRs, most AGDP1-binding sites are in TE regions (Fig. 7a). We found that the average length of AGDP1-associated TEs was greater than the average length of all TEs in the genome (Fig. 7b). Further analysis showed that the enrichment of AGDP1 in long TEs is higher than that in short TEs (Fig. 7c and Supplementary Fig. 10). These results indicate that AGDP1 preferentially binds to long TEs. To investigate the correlation between AGDP1-binding profiles and AGDP1 influence on the methylation of TEs, we determined the relationship between average DNA methylation level and TE length. We found that the absolute levels of CHG and CHH methylation were more affected in long TEs than in short TEs in the *agdp1-1* mutant (Fig. 7d). Based on these results, we conclude that AGDP1 preferentially binds to long TEs and thus affects their DNA methylation.

## Discussion

Our structural studies suggested that the three pairs of tandem AGDs of AGDP1 can recognize a serials histone mark, including unmodified H3R2, H3K4, and H3R8, and dimethylated H3K9. H3K4me0 and H3K9me2 are specifically recognized by the two surface pockets of the AGD1 and AGD2, respectively (Figs. 2a, b). The two pockets are physically adjacent (Figs. 2a, b). However, H3K4me0 and H3K9me2 span six residues, such that the two pockets must be well separated if they are to properly capture both marks. The unique helical conformation of the H3K9me2 peptide can shrink the peptide, making the H3K4me0 and H3K9me2 marks physically closer and fitting into the two adjacent pockets of AGD1 and AGD2, respectively (Figs. 2a, b). Therefore, the unique helical conformation of the peptide ensures the proper and simultaneous capture of the two marks in a dual-recognition mode.

The AGD12 of AGDP1 adopts a tandem tudor-like conformation (Fig. 1f). The tandem tudor has been extensively studied as a methylated histone reader module. Overall, the AGD12 structure resembles the human UHRF1 tandem tudor and *Arabidopsis* SHH1 SAWADEE domains, both of which function as H3K9me readers with similar recognition mechanisms^28,29,36–38^. As shown in Fig. 2h, the SHH1 SAWADEE domain uses the longer edge of the two tudor domain interface to recognize the H3K9me2 peptide, which is long enough to have two distant pockets that can simultaneously accommodate H3K4me0 and H3K9me2 (Fig. 2h). Therefore, the H3K9me2 peptide in the SAWADEE complex adopts an extended conformation (Fig. 2h). In contrast, the AGD12 of AGDP1 uses the shorter edge of the interface between the two AGDs to bind to the H3K9me2 peptide; as a result, the peptide must adopt a condensed conformation to allow the simultaneous positioning of the H3K4me0 and H3K9me2 marks into the two adjacent pockets (Fig. 2h). This mode of binding between AGDP1 and the H3K9me2 peptide has not been observed in other tandem tudor-like structures. Therefore, we conclude that the AGD12–H3K9me2 complex represents a different histone-binding interface on the tandem tudor/AGD-like readers.

Genome-wide mapping of AGDP1-binding sites by ChIP-seq showed that AGDP1 preferentially binds to long TEs. In the *Arabidopsis* genome, short TEs are enriched in chromosome arms, whereas long TEs are enriched in heterochromatin and are marked by H3K9me2^39,40^. Our in vitro binding data suggest that a single AGDP1 protein possesses three H3K9me2-binding sites (Fig. 1e) and sequence alignment results indicate that the AGD12, AGD34, and AGD56 can each capture an H3K9me2 mark using the same mechanism (Fig. 3b). This feature can enhance the binding of AGDP1 to the H3K9me2-enriched heterochromatin region, providing the biochemical basis for our observation of the enrichment of AGDP1 in the H3K9me2-associated long TEs.

Although AGD was identified in 2003^20^, the functional significance of this domain has remained unclear. Here, our structural studies reveal that the tandem AGDs of AGDP1 can specifically recognize the methylated K9 and unmethylated K4 on the H3 tail through two negatively charged surface pockets, structurally defining the AGD as a histone mark reader. We also demonstrated that AGDP1 is associated with heterochromatin and is required for transcriptional silencing, non-CG DNA methylation, and H3K9 dimethylation at some loci. The hypo-DMRs of *agdp1* overlap with those of *suvh4/5/6* and *cmt2/3*, indicating that AGDP1 is functionally involved in the cyclic feedback loop between H3K9me2 and non-CG methylation. Both SUVH4/5/6 and CMT2/3 can directly couple the reading of an epigenetic silencing mark and the writing of another epigenetic silencing mark by themselves^13,16^. To identify AGDP1-associated factors, we performed immunoprecipitation followed by mass spectrometry (IP-MS) analysis with *pAGDP1-Flag-AGDP1* transgenic plants for four replicates, but did not identify SUVH4/5/6, CMT2/3, and DDM1 from the Flag-AGDP1 IP samples (Supplemental Data 6). It is possible that AGDP1 does not directly recruit SUVH4/5/6, CMT2/3, or DDM1 but instead serves in a structural role, such as acting as a scaffold protein to bridge multiple nucleosome together. Our ChIP-seq data indicated that AGDP1 is preferentially located at long TEs. Previous studies showed that DDM1 allow methyltransferase access to nucleosomal templates and longer TEs that are blocked by the H1 linker^40,41^. It is still unknown why DDM1 preferentially affects the chromatin architecture of long TEs but not short TEs. We found a substantial overlap of hypo-DMRs between *agdp1* and *ddm1* mutants (Supplementary Fig. 4). We propose that the binding of AGDP1 to the heterochromatin mark H3K9me2 might induce a change in chromatin structure and thereby create a permissive chromatin environment for DDM1 and subsequently for the DNA methyltransferases. It would be interesting to see if histone *h1* mutation can suppress *agdp1* mutant phenotype by future studies. The enrichment of AGDP1 within the nucleolus indicates that AGDP1 may also have functions related to the nucleolus. The nucleolus is the site of 45S pre-rRNA transcription, rRNA, tRNA, and endogenous nuclear siRNAs processing^42,43^. The preferential binding of AGDP1 to heterochromatin is consistent with the nucleolus-associated chromatin domains in *A. thaliana* being primarily genomic regions with heterochromatic signatures. These regions also include TEs and mostly inactive protein-coding genes^44^. Further studies are required to clarify the functions of AGDP1 in the nucleolus.

## Methods

### Plant materials and growth conditions

All plants were grown under a long-day photoperiod with 16 h of light at 22 °C and 8 h of darkness at 20 °C. The *Arabidopsis* T-DNA insertion lines *agdp1-1* (Salk_134878) and *agdp1-2* (Salk_130936) were obtained from the Arabidopsis Biological Resource Center. The *suvh4/5/6* mutant was obtained from J. Bender, Brown University^11^.

To generate *AGDP1* complementation lines, the 2172-bp promoter regions of *AGDP1* and *AGDP1* genomic DNA were cloned into the modified *pCAMBIA1305* vector (with a 3×Flag tag at the N terminus of AGDP1) using the In-Fusion HD Cloning Kit (Clontech, 639648). The construct was transformed into *agdp1-1* mutants using *Agrobacterium* GV3101 and the standard floral dip method. The primers used for plasmids construction are listed in Supplementary Data 7.

### Peptide affinity purification and mass spectrometric analysis

To extract the nuclear proteins for peptide affinity purification, 1 g of wild-type *Arabidopsis* flowers was harvested and ground in liquid nitrogen. The ground material was homogenized in 15 ml of Honda Buffer (20 mM HEPES-KOH, pH 7.4, 0.44 M sucrose, 1.25% ficoll, 2.5% Dextran T40, 10 mM MgCl_2_, 0.5% Triton X-100, 5 mM DTT, 1 mM phenylmethylsulfonyl fluoride (PMSF), Roche protease inhibitor cocktail) and filtered through two layers of Miracloth. The filtrates were centrifuged at 2000 *g* for 15 min at 4 °C to precipitate the nuclei. Nuclear pellets were washed two times with 1 ml of Honda buffer and then were resuspended in 600 μl of Nuclei Lysis Buffer (50 mM Tris-HCl pH 8.0, 150 mM NaCl, 1% NP40, 0.5 mM DTT, 1 mM PMSF, Roche protease inhibitor cocktail). After sonication, nuclei extracts were centrifuged at 20,000 *g* for 10 min at 4 °C. The supernatant was incubated with 2 μg of biotinylated histone peptides and Streptavidin Magnetic Beads (Thermo Scientific, 88816) for 3 h with rotation at 4 °C. Histone peptides H3 (12-403), H3K9me1 (12-569), H3K9me2 (12-430), and H3K9me3 (12-568) were purchased from EMD Millipore. After incubation, beads were washed three times with wash buffer 1 (320 mM NaCl, 50 mM Tris-HCl pH 8.0, 0.5% NP40), and three times with wash buffer 2 (150 mM NaCl, 50 mM Tris-HCl pH 8.0).

For LC-MS/MS analysis, IP beads with captured proteins were lysed in 12 mM sodium deoxycholate (SDC)-sodium lauroyl sarcosinate (SLS): 100 mM Tris-HCl (pH 8.5), 12 mM SDC, and 12 mM SLS. Proteins were reduced and alkylated with 10 mM tris-(2-carboxyethyl) phosphine and 40 mM chloroacetamide at 95 °C for 5 min. The supernatant containing reduced and alkylated proteins was transferred into a new Eppendorf tube and diluted fivefold with 50 mM triethylammonium hydrogen carbonate buffer and digested with 500 ng trypsin (Sigma) overnight. The SDC and SLS were removed by adding 100% acetyl acetate in a ratio 1:1 (vol/vol) sample to acetyl acetate volume ratio. Samples were acidified with 10% trifluoroacetic acid to pH ~3, and desalted by a custom-made stage tip with a styrene divinyl benzene (SDB-XC) membrane (3 M). The dried peptides were resuspended in 30 μL of 0.3% formic acid (FA) with 3% acetonitrile (ACN), quantified by BCA assay (Thermo Fisher Scientific), and injected into an Easy-nLC 1000 (Thermo Fisher Scientific). Peptides were separated on a 45 cm in-house packed column (360 μm OD × 75 μm ID) containing C18 resin (2.2 μm, 100 Å, Michrom Bioresources) with a 30 cm column heater (Analytical Sales and Services) set at 60 °C. The mobile phase buffer consisted of 0.1% FA in ultra-pure water (buffer A) with an eluting buffer of 0.1% FA in 80% ACN (buffer B) run over a linear 65 min gradient of 5%-30% buffer B at a flow rate of 250 nL/min. The Easy-nLC 1000 was coupled online with a Velos Pro LTQ-Orbitrap mass spectrometer (Thermo Fisher Scientific). The mass spectrometer was operated in the data-dependent mode in which a full MS scan (from m/z 350–1500 with the resolution of 30,000 at 400 m/z) was followed by the 10 most intense ions being subjected to collision-induced dissociation (CID) fragmentation. CID fragmentation was performed and acquired in the linear ion trap (normalized collision energy 30%, AGC 3e4, max injection time 100 ms, isolation window 3 m/z, and dynamic exclusion 60 s). The raw files were searched directly against *Arabidopsis thaliana* database (TAIR10) with no redundant entries using SEQUEST HT algorithm in Proteome Discoverer version 2.2 (Thermo Fisher Scientific). Peptide precursor mass tolerance was set at 15 ppm, and MS/MS tolerance was set to 0.6 Da. Search criteria included a variable modifications of oxidation ( + 15.995 Da) on methionine residues, and acetylation ( + 42.011 Da) at N-terminus of protein, and a static carbamidomethylation of cysteines ( + 57.021 Da). Search was performed with full tryptic digestion and allowed a maximum of two missed cleavages on the peptides analyzed from the sequence database. Relaxed and strict false discovery rates were set for 0.05 and 0.01, respectively.

### Protein expression and purification

The gene encoding AGD12 of RsAGDP1 (residues 26–180) was cloned into a self-modified pMBP-His vector to fuse N-terminal hexahistidine plus an MBP tags to the target protein. The plasmids were transformed into *Escherichia coli* strain BL21(DE3) RIL. When the OD600 of the cell culture reached 0.7, expression of the protein was induced at 20 °C by adding isopropyl β-D-1-thiogalactopyranoside to a final concentration of 0.2 mM. The recombinant expressed protein was purified by a HisTrap column (GE Healthcare). The His-MBP tag was cleaved by tobacco etch virus protease and removed by a second step HisTrap column (GE Healthcare). The target protein was further purified using a Superdex G200 gel filtration column (GE Healthcare). AtAGDP1 AGD12 (residues 12–183), AGD34 (residues 198–364), AGD56 (residues 375–517), full-length AtAGDP1, RsAGDP1 AGD34 (residues 201–350), and AGD56 (residues 361–508) were cloned, expressed, and purified using the same protocol as used for RsAGDP1 AGD12. Because the RsAGDP1 AGD12 has no methionine residues, an L54M/L135M/L140M triple mutant was generated for producing Se-Met-substituted proteins. The Se-Met-substituted proteins were expressed in Se-Met containing M9 medium and were purified using the same protocol as used for the wild-type protein. All mutations were generated using a PCR-based method and were expressed and purified using the same protocol as used for the wild-type proteins. The peptides used in this study were purchased from the GL Biochem (Shanghai) Ltd.

### Crystallization and structure determination

Before crystallization, the RsAGDP1 AGD12 was mixed with an H3(1-15)K9me2 peptide with a molar ratio of 1:4. The Se-Met-labeled RsAGDP1 AGD12 L54M/L135M/L140M triple mutant in complex with an H3K9me2 peptide was crystallized in a condition of 15% PEG20000, 0.1 M MES, pH 6.5. The Se-Met-labeled AtAGDP1 AGD34 was crystallized in a condition of 3.5 M sodium formate, 0.1 M HEPES, pH 7.5. The crystals were cryo-protected in the reservoir solution supplemented with 15% glycerol and were flash cooled in liquid nitrogen. All data were collected at the beamline BL18U1 of the National Center for Protein Sciences Shanghai (NCPSS) at the Shanghai Synchrotron Radiation Facility (SSRF). The data were processed using the program HKL3000 suite^45^. The structure of RsAGDP1 AGD12-H3K9me2 complex was determined using the single-wavelength anomalous dispersion (SAD) method as implemented in the program Phenix^46^. The structure refinement and manual model building were carried out using the programs Phenix and Coot, respectively^46,47^. Throughout the refinement, the geometry of the structure model was monitored using the program Molprobity^48^. The structure of AtAGDP1 AGD34 in free from was determined using the SAD method, too. The statistics for data collection and structure refinement are summarized in Supplementary Table 1.

### ITC

The ITC-based binding experiments were carried out on a Microcal PEAQ-ITC instrument (Malvern) at 25 °C. The protein samples were dialyzed overnight at 4 °C. The protein samples were then diluted to 0.1 mM, and the lyophilized peptides were dissolved with the same buffer with a final concentration of 1.0–1.5 mM. The titration was performed with the standard protocol, and the data were fit using the program Origin 7.0.

### RNA sequencing and data analysis

We performed RNA deep sequencing assay with five replicates for wild-type and *agdp1-1*, and three replicates for *suvh4/5/6*. Total RNA was extracted from 0.2 g of 10-day-old *Arabidopsis* seedlings with Trizol reagent (Invitrogen) and was sent to the Genomics Core Facilities of the PSC (Shanghai Center for Plant Stress Biology, Shanghai, China) for library preparation according to the manufacturer’s (Illumina) instructions. The libraries were sequenced on an Illumina Hiseq 2500 platform. Reads were mapped to the TAIR10 genome using TopHat2^49^ with parameter “--b2-very-sensitive”. The differential expression of protein-coding genes and TEs was identified with “Cuffdiff” program (with parameter “-u -b < genome.fa > ”) of Cufflinks^50^ (*P* < 0.05; log2(fold-change) > 1).

### WGBS and data analysis

Genomic DNA was extracted from 10-day-old seedlings using the DNeasy Plant Maxi Kit (Qiagen, 68163) and was sent to the Genomics Core Facilities of the PSC (Shanghai Center for Plant Stress Biology, Shanghai, China) for bisulfite treatment. Libraries were prepared for sequencing according to the manufacturer’s (Illumina) instructions and were sequenced on an Illumina HiSeq 2500 platform. For data analysis, low-quality sequences (*q* < 20) were trimmed using trim in BRAT-BW^51^, and clean reads were mapped to the TAIR10 genome using BRAT-BW and allowing two mismatches. To remove potential PCR duplicates, the remove-dupl command of BRAT-BW was used. DNA hypomethylated regions were identified according to Stroud et al.^15^ with some modifications. In brief, the genome was divided into 100-bp bins. Fisher’s exact test was performed for methylated versus unmethylated cytosines for each context, within each bin, with false discovery rates (FDRs) estimated using a Benjamini–Hochberg (BH) adjustment of Fisher’s *P*-values calculated in the R environment. Bins with an absolute methylation difference of 0.4, 0.2, and 0.1 for CG, CHG, and CHH, respectively, and with a BH-corrected FDR < 0.01 were selected. To avoid 100-bp bins with few cytosines, only bins with at least four corresponding cytosines that were covered by at least four reads in both the mutant and wild-type were retained. The WGBS experiments were performed with one replicate and the reduction of DNA methylation was confirmed by chop-PCR. Full scans of the agarose gels and western blots are shown in Supplementary Fig. 11.

### ChIP assays

ChIP was performed following the instruction from a previously published study with minor modifications^52^. In brief, 1 g of 10-day-old seedlings were cross-linked with 1% formaldehyde for 20 min under vacuum and ground into fine powder in liquid nitrogen. Chromatin was isolated and sheared into 200- to 500-bp fragments by sonication. The sonicated chromatin was incubated with 2 μg of anti-H3K9me2 (Abcam, ab1220), anti-H3 (Abcam, ab1791), or anti-Flag (Sigma, F1804) antibody and with 25 μl of Dynabeads Protein G (Invitrogen, 10003D) for 5 h with rotation at 4 °C. The precipitated chromatin DNA was then recovered and purified with a standard phenol–chloroform method. ChIP-qPCR was performed and results were calculated as percentage of input DNA. Sequences for the primers used for ChIP-qPCR are listed in Supplementary Data 7.

### ChIP-seq and data analysis

Two biological replicates were prepared and sequenced for each ChIP-seq experiment. For ChIP-seq, DNA from three ChIPs was pooled to ensure enough DNA for library construction. ChIP samples were sent to the Genomics Core Facilities of the PSC (Shanghai Center for Plant Stress Biology, Shanghai, China) for library construction and Illumina sequencing. ChIP-seq reads were mapped to the TAIR10 genome with Bowtie 2^53^ with parameter “-k 10 --very-sensitive --no-unal --no-mixed --no-discordant” and PCR duplicates were removed with “samtools rmdup”^54^. AGDP1-binding peaks were identified with SICER^55^ (parameter: [“redundancy threshold” = 1] [“window size (bp)” = 200] [“fragment size” = 200] [“effective genome fraction” = 0.84] [“gap size (bp)” = 600] [“FDR” = 0.05]). AGDP1 peaks associated histone features were analyzed as previously described^35^. The random peaks with the same length distribution is generated by “shuffle” command from the BEDTools suite.

### AGDP1 nuclear localization

*35S::GFP* was cloned into the modified *pCAMBIA1305* vector, which was named *pCAMBIA1305-35S::GFP*. To determine the nuclear localization of AGDP1, the coding sequence of *AGDP1* was amplified and cloned into the *pCAMBIA1305-35S::GFP* vector with its N-terminal tagged by GFP. Leaves of tobacco plants (*N. benthamiana*) were infiltrated with *agrobacterium* GV3101 strain carrying *pCAMBIA1305-35S::GFP* or *pCAMBIA1305-35S::GFP-AGDP1* plasmids. After 2 days, GFP-AGDP1 and GFP control fluorescence were observed in leaves of tobacco plants with a Zeiss LSM 880 upright confocal microscope.

### Analysis of RNA transcripts at individual loci

Total RNA was extracted from 10-day-old seedlings with Trizol reagent (Invitrogen, 15596026). After contaminated DNA was removed by the DNA-free Kit (Invitrogen, AM1907), 1 μg of total RNA was used for RT using both oligo dT and random primers. Real-time PCR was performed on a CFX96 real-time PCR detection system (Bio-Rad) using iTaq Universal SYBR Green Supermix (Bio-Rad, 1725122). The actin gene *ACT7* was amplified as an internal control. The results presented were based on at least three biological replications. All primer sequences are listed in Supplementary Data 7.

## Electronic supplementary material

Supplementary Information

Peer Review File

Description of Additional Supplementary Files

Supplementary Data 1

Supplementary Data 2

Supplementary Data 3

Supplementary Data 4

Supplementary Data 5

Supplementary Data 6

Supplementary Data 7

## Data Availability

All high-throughput sequencing data generated in this study have been deposited in GEO with accession codes GSE111609. X-ray structures have been deposited in the RCSB Protein Data Bank with accession codes 5ZWX for RsAGDP1 AGD12-H3K9me2 complex and 5ZWZ for AtAGDP34. All other data are available from the corresponding authors on request.
