# Supplementary material for: Arabidopsis AGDP1 links H3K9me2 to DNA methylation in heterochromatin
Source: Nat Commun. 2018 Oct 31;9:4547. doi: 10.1038/s41467-018-06965-w (PMC6208443; doi:10.1038/s41467-018-06965-w)
Supplement: Supplementary file 1 — Supplementary Information [file 41467_2018_6965_MOESM1_ESM.pdf]

Arabidopsis AGDP1 links H3K9me2 to DNA methylation in heterochromatin

Zhang et al.

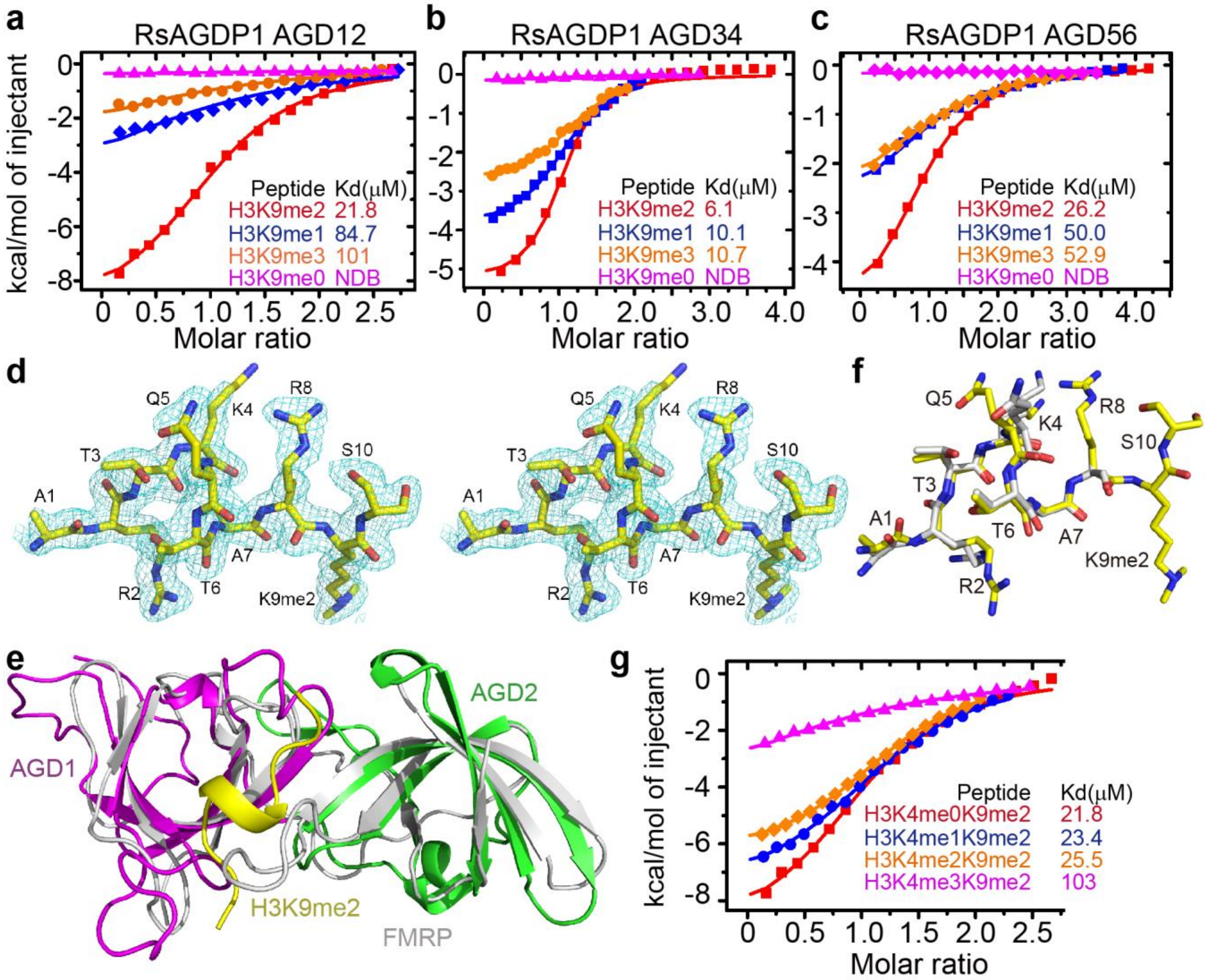
a
RsAGDP1 AGD12
kcal/mol of injectant
Molar ratio
Peptide Kd(μM)
H3K9me2 21.8
H3K9me1 84.7
H3K9me3 101
H3K9me0 NDB
b
RsAGDP1 AGD34
Molar ratio
Peptide Kd(μM)
H3K9me2 6.1
H3K9me1 10.1
H3K9me3 10.7
H3K9me0 NDB
c
RsAGDP1 AGD56
Molar ratio
Peptide Kd(μM)
H3K9me2 26.2
H3K9me1 50.0
H3K9me3 52.9
H3K9me0 NDB
d
A1 R2 T3 K4 Q5 T6 A7 R8 K9me2 S10
f
A1 R2 T3 K4 Q5 T6 A7 R8 K9me2 S10
e
AGD1
AGD2
H3K9me2
FMRP
g
kcal/mol of injectant
Molar ratio
Peptide Kd(μM)
H3K4me0K9me2 21.8
H3K4me1K9me2 23.4
H3K4me2K9me2 25.5
H3K4me3K9me2 103

Supplementary Figure 1. AGDP1 recognizes H3K9me2 mark. a-c ITC binding curves between different methylated H3K9 peptides and AGD12 (**a**), AGD34 (**b**), and AGD56 (**c**) of RsAGDP1 show that all three tandem-AGD cassettes recognize methylated H3K9 marks with a preference on H3K9me2, which is similar as AtAGDP1 tandem AGDs. NDB, no detectable binding. **d** A stereo view of the SIGMAA weighted 2Fo-Fc electron density map of the H3K9me2 peptide at 1 s level is shown in cyan mesh with the peptide in stick representation. **e** The superimposition of the RsAGDP1 AGD12-H3K9me2 complex with the FMRP tandem-AGD (PDB code: 4QVZ) showing similar overall structures. **f** The superimposition of the H3K9me2 peptide from the RsAGDP1 AGD12-H3K9me2 complex in yellow and the unmodified H3 peptide from the BAZ2A PHD-H3 complex (PDB code: 5T8R) in silver reveals an almost identical helical conformation. **g** ITC binding curves between RsAGDP1 AGD12 and H3K4me0/1/2/3K9me2 peptides show that the increase of the H3K4 methylation status can decrease the binding between AGD12 and histone peptides.

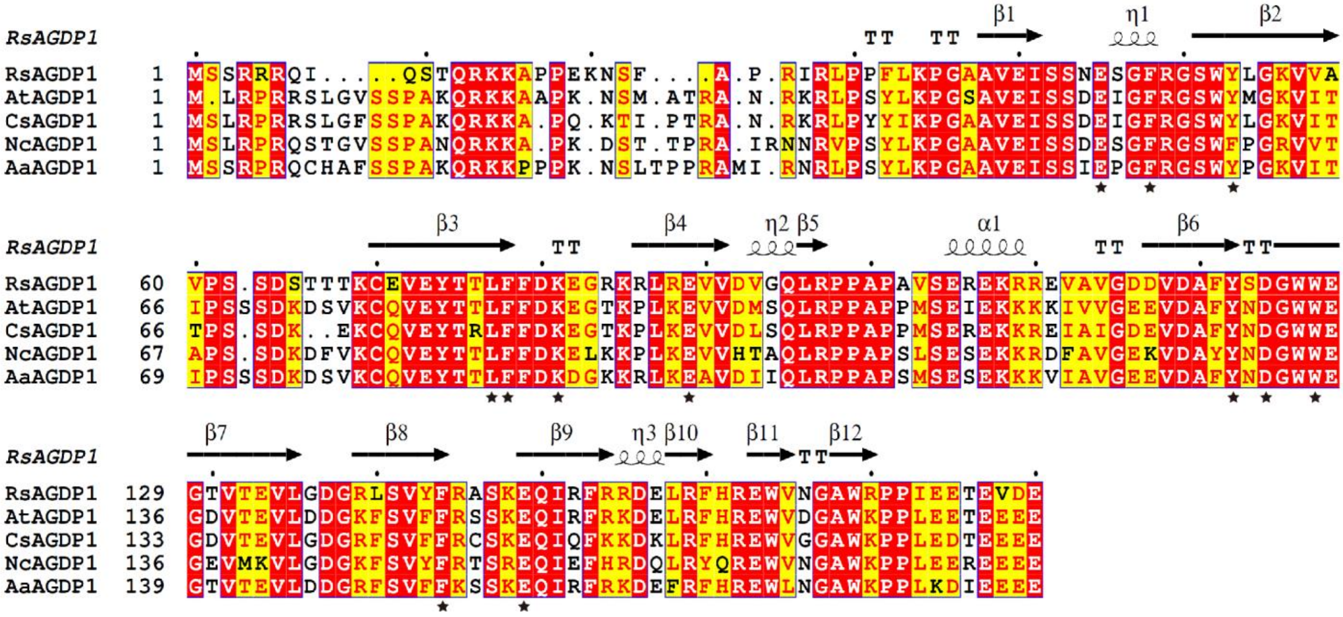

Supplementary Figure 2. A structure-based sequence alignment of the AGD12 of AGDP1 from difference species. The secondary structures RsAGDP1 AGD12 is labeled on the top of the alignment. The residues involving in H3K9me2 peptide recognition are strictly conserved and marked by stars. The sequence can be found in NCBI with accession codes of: XP_018485729.1 (*Raphanus sativus* AGDP1, RsAGDP1), NP_172403.3 (*Arabidopsis thaliana* AGDP1, AtAGDP1), XP_010475764.1 (*Camelina sativa* AGDP1, CsAGDP1), JAU60727.1 (*Noccaea caerulescens* AGDP1, NcAGDP1), and KFK43229.1 (*Arabis alpine* AGDP1, AaAGDP1).

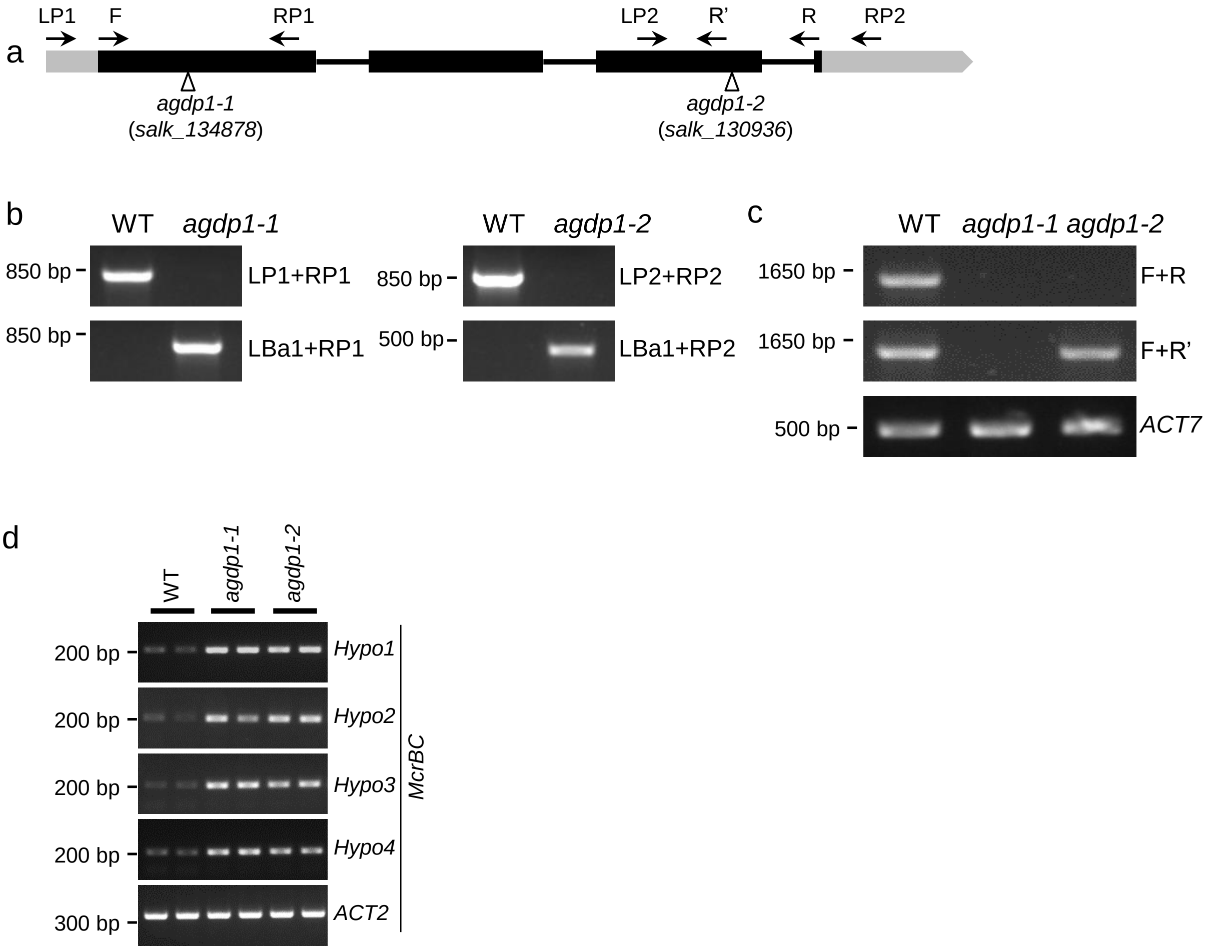
a
LP1
F
RP1
LP2
R'
R
RP2
agdp1-1
(salk_134878)
agdp1-2
(salk_130936)
b
WT
agdp1-1
850 bp
LP1+RP1
850 bp
LBa1+RP1
WT
agdp1-2
850 bp
LP2+RP2
500 bp
LBa1+RP2
c
WT
agdp1-1
agdp1-2
1650 bp
F+R
1650 bp
F+R'
500 bp
ACT7
d
WT
agdp1-1
agdp1-2
200 bp
Hypo1
200 bp
Hypo2
200 bp
Hypo3
200 bp
Hypo4
300 bp
ACT2
McrBC

Supplementary Figure 3. Characterization of T-DNA insertion mutations in *AGDP1.*

a, Diagram showing the *AGDP1* gene and salk mutants T-DNA insertion sites. Gray and black boxes represent *AGDP1* gene untranslated regions and exons, respectively. Black lines indicate introns of *AGDP1* gene. The arrows represent PCR primers used in genotyping and RT-PCR analysis.

b, Genotyping analysis of *agdp1* T-DNA mutant alleles.

c, RT-PCR analysis of AGDP1 gene expression in the wild type and *agdp1* mutants.

d, DNA methylation of hypo-DMRs were evaluated by chop-PCR in wild type, *agdp1-1* and *agdp1-2*. *ACT2* was amplified as an internal control.

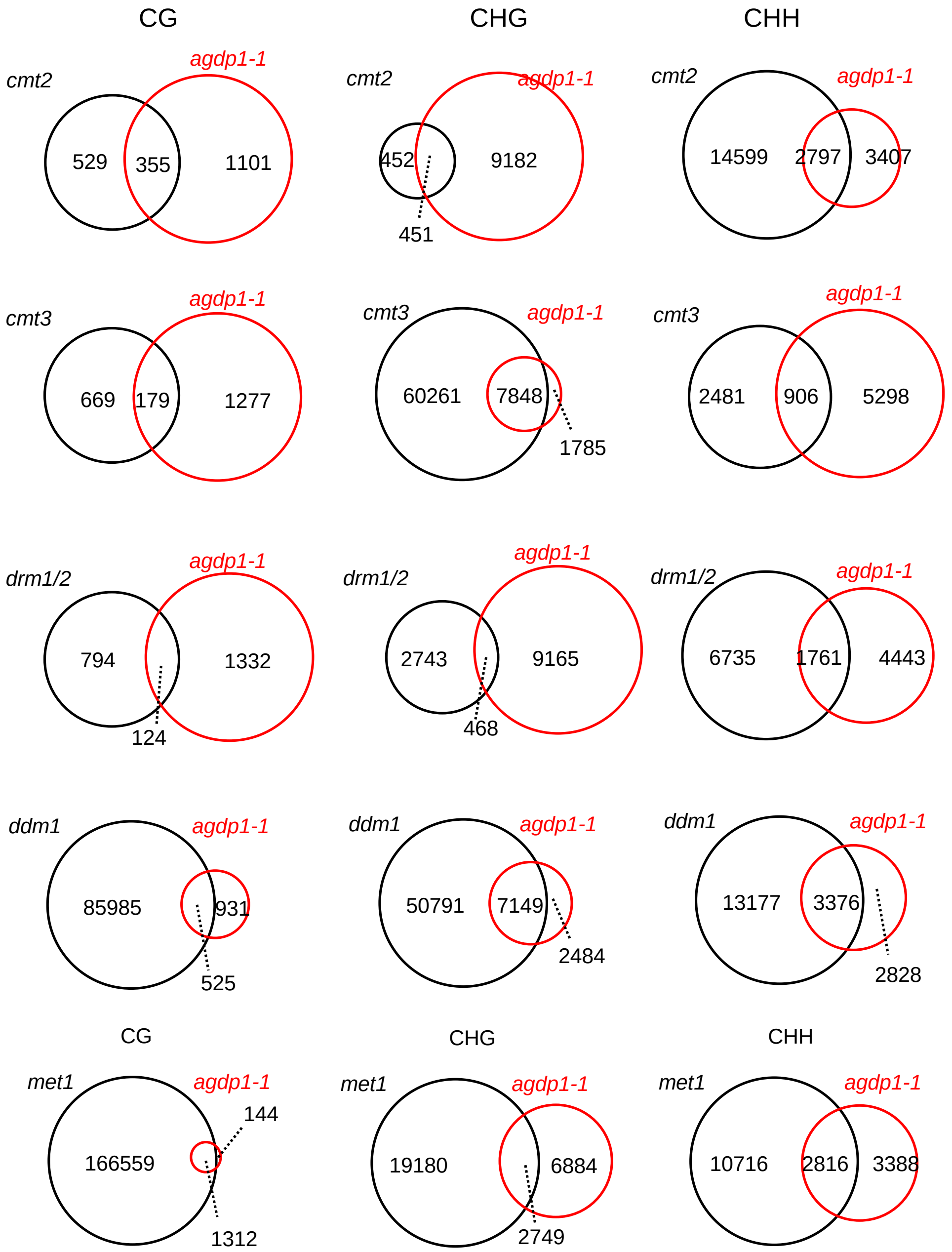

Supplementary Figure 4. Overlap analysis of hypo-DMRs between *agdp1-1* and the indicated mutants in CG, CHG and CHH methylation.

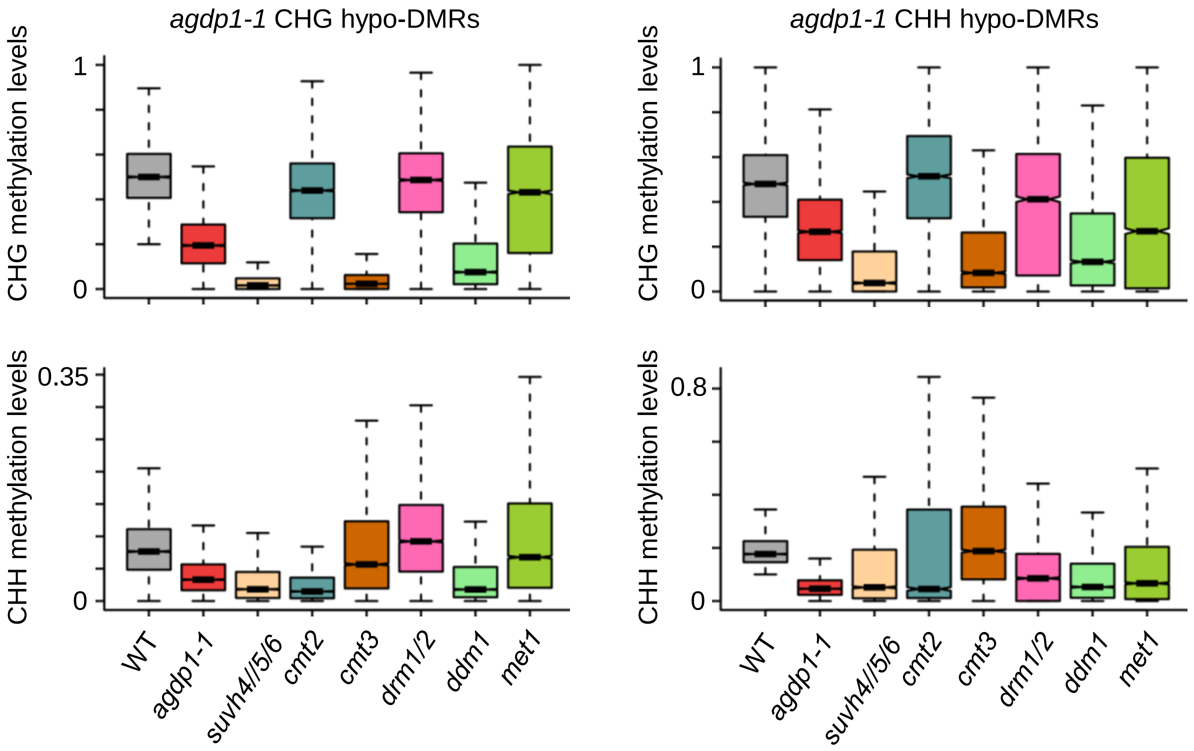

Supplementary Figure 5. Boxplots represent CHG and CHH methylation levels of *agdp1-1* CHG and CHH hypo-DMRs in the indicated mutants.

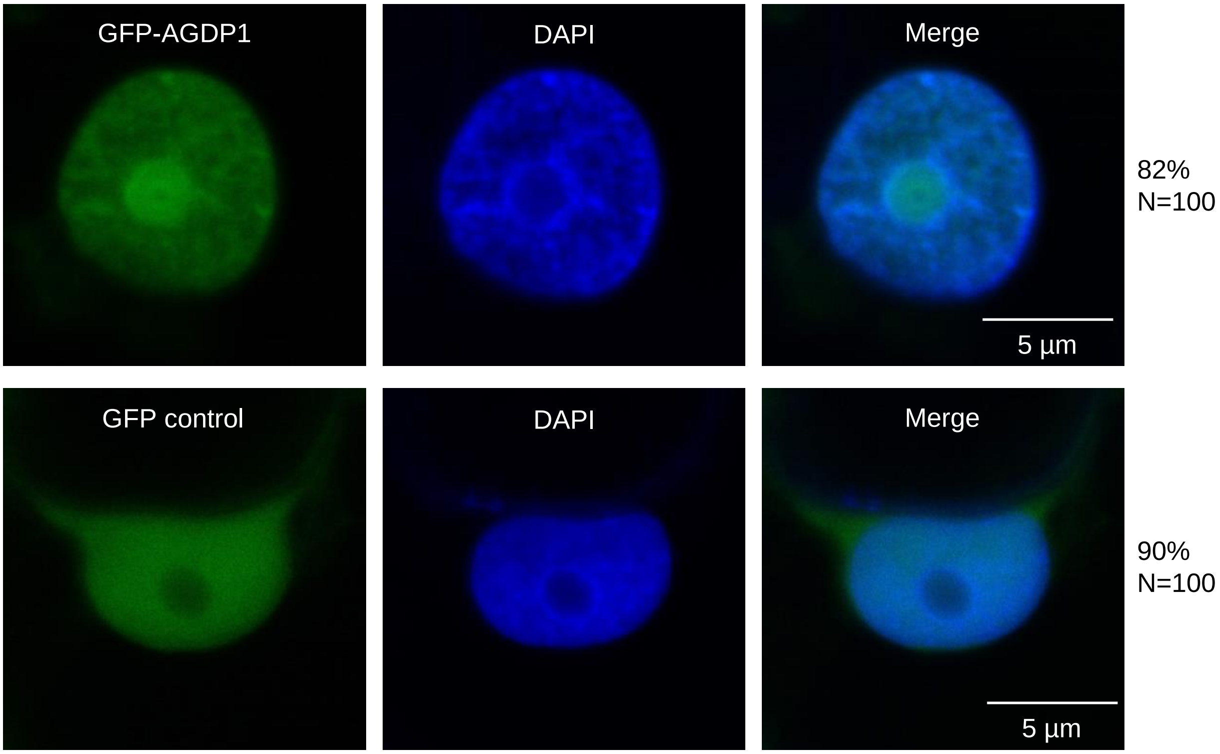

Supplementary Figure 6. Subnuclear localization of AGDP1.

Confocal microscopy images show subnuclear localization of GFP-AGDP1 (top panels) and GFP (bottom panels) in *N. benthamiana*. Nuclei were counterstained with DAPI (blue). The percentage of the nuclei with indicated patterns is shown.

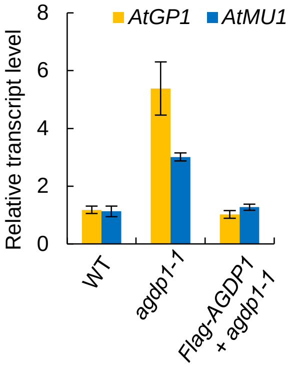

Supplementary Figure 7. Quantitative RT–PCR analyses showing increased expression of *AtGP1* and *AtMU1* in *agdp1-1* mutant can be rescued by p*AGDP1*::*Flag-AGDP1* transgene.

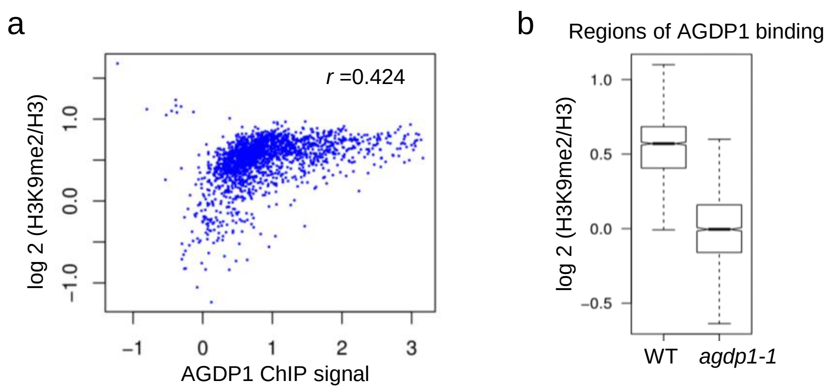

Supplementary Figure 8. AGDP1 enrichment is associated with H3K9me2 modification.

a, The association between H3K9me2 and AGDP1 enrichment was analyzed by scatter plot. The Pearson correlation coefficient is shown.

b, Box plots of levels of H3K9me2 relative to H3 in AGDP1 binding regions.

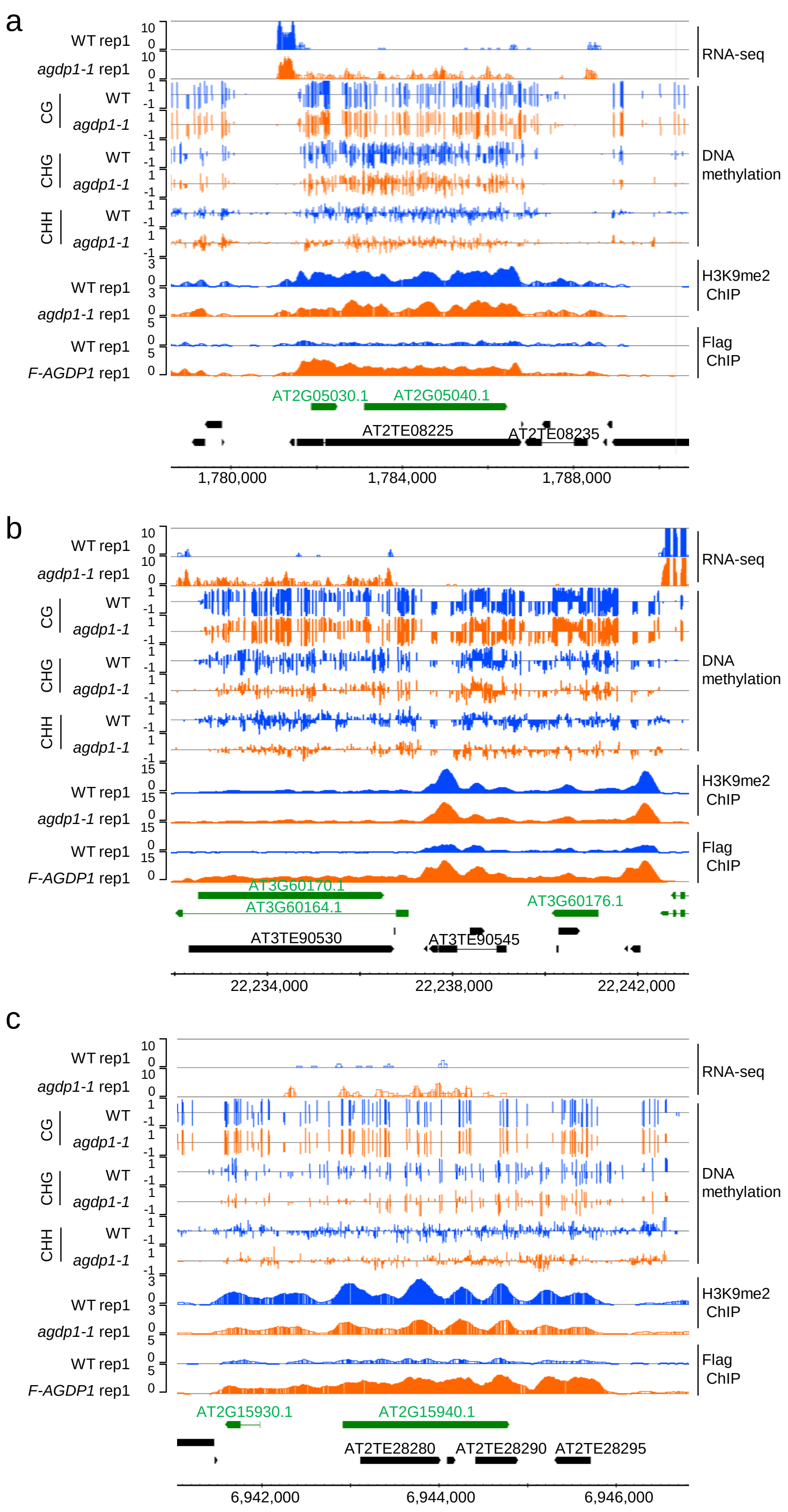

Supplementary Figure 9. Snapshots showing RNA expression, DNA methylation, H3K9me2 levels and AGDP1 enrichment at three representative regions. The y axis is normalized read counts per million (ChIP-seq) or ten million (RNA-seq). The normalization factor is calculated by the total mapped reads.

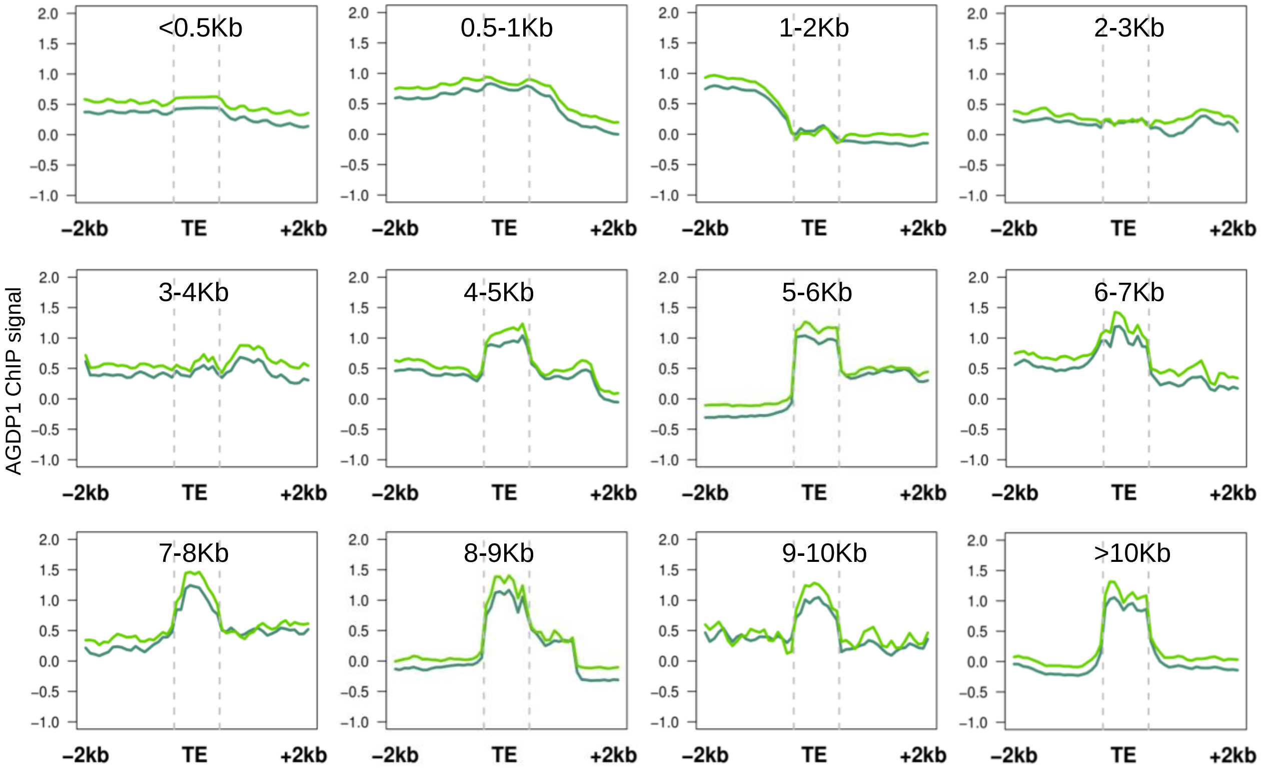

Supplementary Figure 10. AGDP1 preferentially binds to long TEs.

Metaplot of AGDP1 enrichment along different length of TEs. AGDP1 ChIP signal was showen as log 2 (AGDP1 ChIP / Input). Dark green and light green curves represent ChIP signals of replicate 1 and 2, respectively.

Supplementary Figure 11. Full-size images of western blots and gels shown in this study.

Fig. 5b

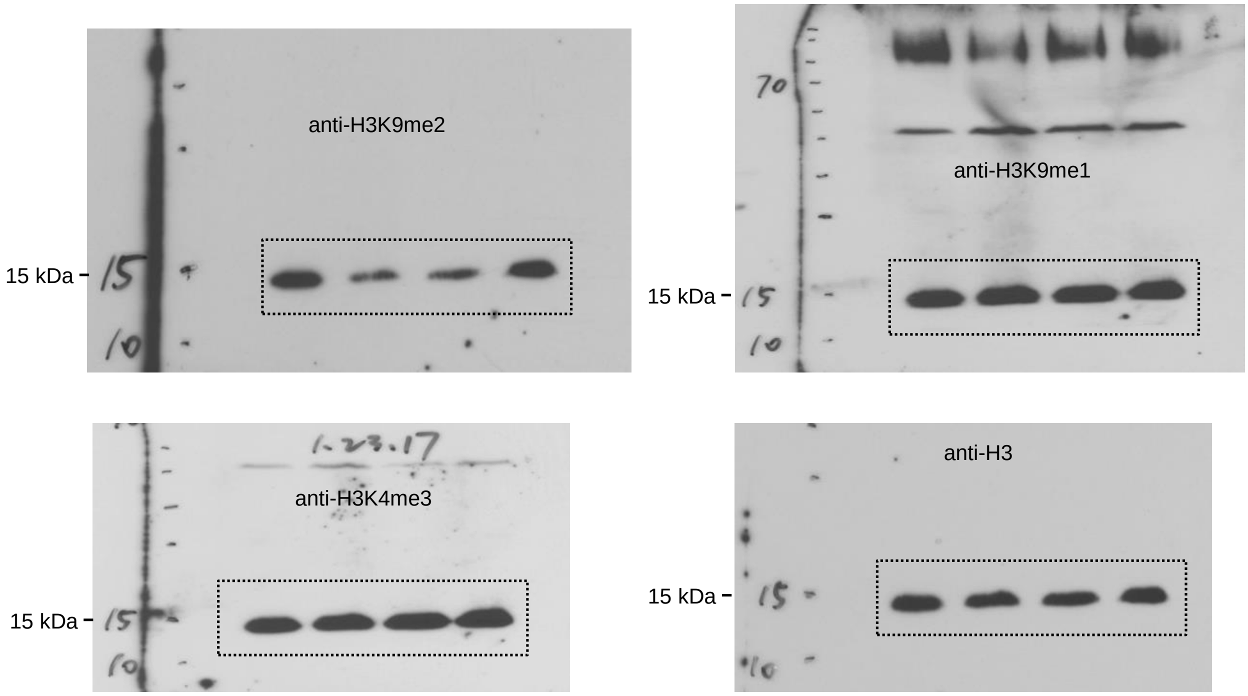

Supplementary Fig. 3b

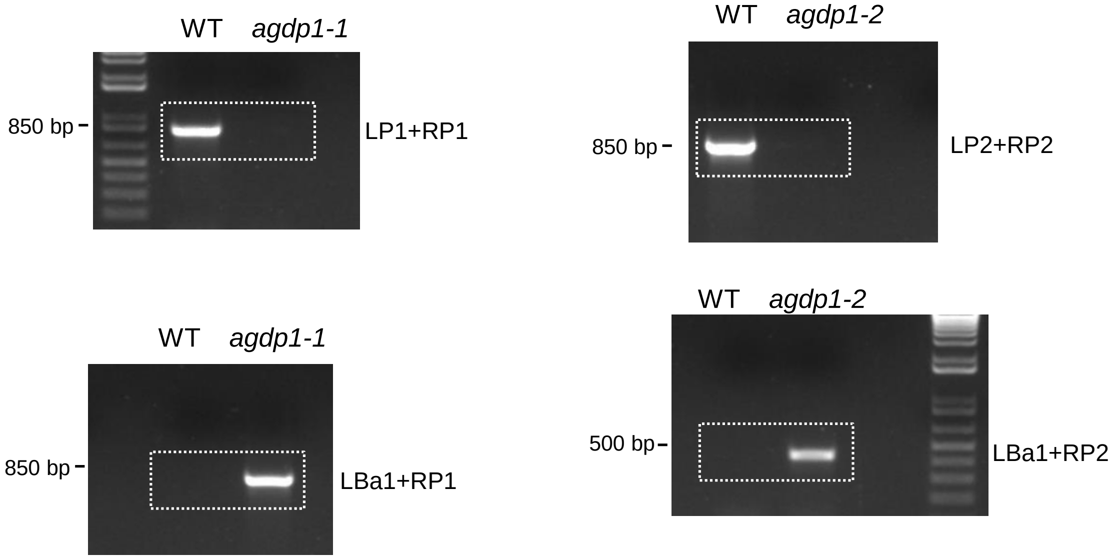

Supplementary Fig. 3c

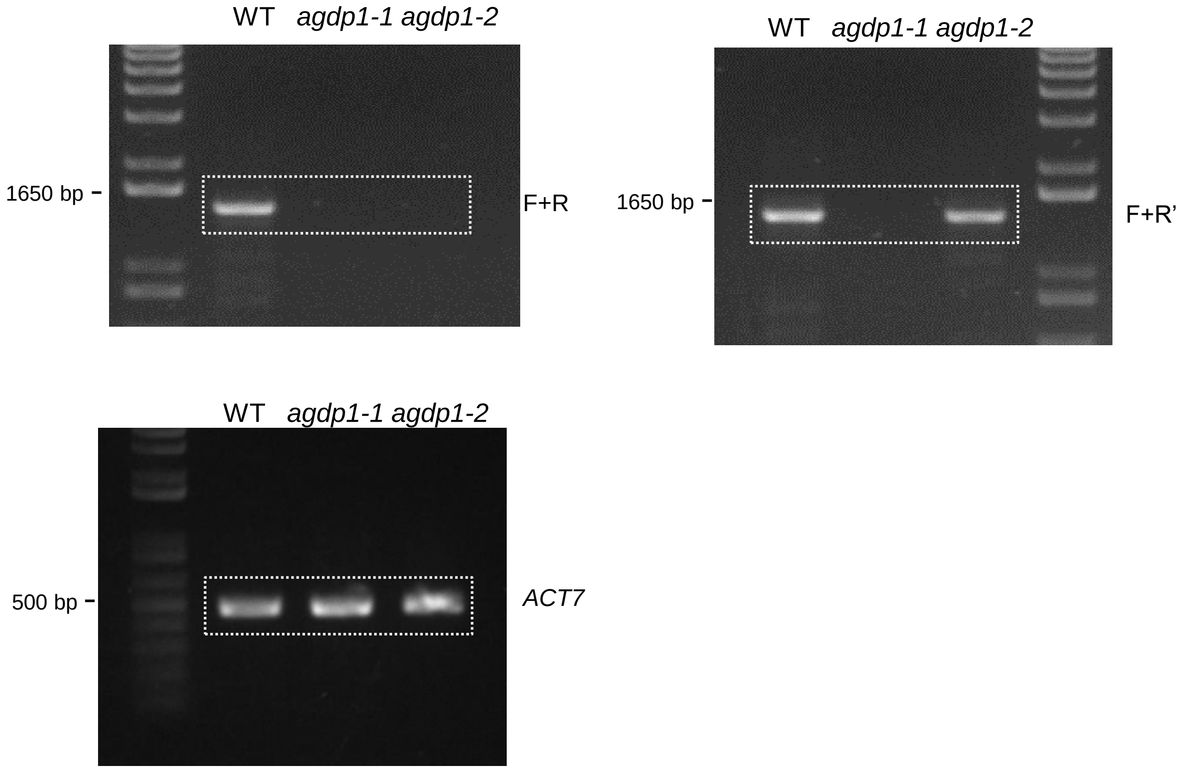

Supplementary Fig. 3d

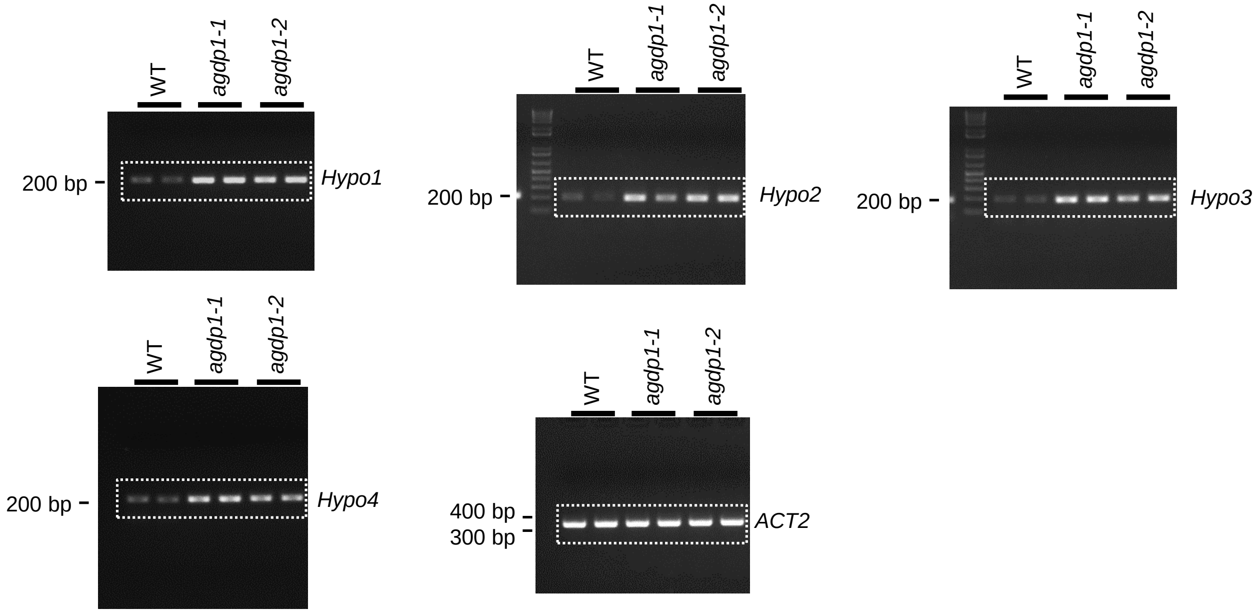

Supplementary Table 1. Data collection and refinement statistics

	RsAGDP1-AGD12-H3K9me2	AtAGDP1-AGD34
**Data collection**		
Beamline	SSRF-BL18U1	SSRF-BL18U1
PDB code	5ZWX	5ZWZ
Space group	$P2_1$	$P6_522$
Wavelength (Å)	0.9778	0.9778
Cell dimensions		
a, *b*, *c* (Å)	47.7, 55.0, 83.6	57.2, 57.2, 206.4
α, β, γ (°)	90, 99.7, 90	90, 90, 120
Resolution (Å)	50.0-1.9 (1.97-1.90)[*]	50.0-2.0 (2.07-2.00)
R_{merge}	0.082 (0.567)	0.097 (0.241)
$I / \sigma I$	21.5 (2.5)	56.0 (17.8)
Completeness (%)	99.7 (99.6)	99.4 (98.6)
Redundancy	6.7 (6.8)	15.6 (17.8)
**Refinement**		
No. reflections	33,643	14,371
R_{work} / R_{free}	0.182 / 0.211	0.200 / 0.219
No. atoms	2,814	1,272
Protein / Peptide	2,404 / 162	1,185 / -
Water	248	87
B-factors (Å^2)	47.5	51.8
Protein / Peptide	47.8 / 41.1	51.8 / -
Water	48.9	52.5
RMS deviations		
Bond lengths (Å)	0.012	0.004
Bond angles (°)	1.422	0.845

[*]Highest-resolution shell is shown in parentheses.
