# Supplementary material for: Arabidopsis AGDP1 links H3K9me2 to DNA methylation in heterochromatin
Source: Nat Commun. 2018 Oct 31;9:4547. doi: 10.1038/s41467-018-06965-w (PMC6208443; doi:10.1038/s41467-018-06965-w)
Supplement: Supplementary file 3 — Description of Additional Supplementary Files [file 41467_2018_6965_MOESM3_ESM.pdf]

Description of Additional Supplementary Files

File Name: Supplementary Data 1
Description: List of proteins identified through peptide affinity purification.

File Name: Supplementary Data 2
Description: RNA transcript levels of differentially expressed protein coding genes and TEs.

File Name: Supplementary Data 3
Description: DMRs identified in *agdp1-1*.

File Name: Supplementary Data 4
Description: Classification analysis for TE families.

File Name: Supplementary Data 5
Description: List of Flag-AGDP1 enriched peaks.

File Name: Supplementary Data 6
Description: List of proteins identified by mass spectrometric analyses in affinity purification of Flag-AGDP1.

File Name: Supplementary Data 7
Description: List of primers used in this study.
