# Supplementary material for: Arabidopsis AGDP1 links H3K9me2 to DNA methylation in heterochromatin
Source: Nat Commun. 2018 Oct 31;9:4547. doi: 10.1038/s41467-018-06965-w (PMC6208443; doi:10.1038/s41467-018-06965-w)
Supplement: Supplementary file 2 — Peer Review File [file 41467_2018_6965_MOESM2_ESM.pdf]

Reviewer #1 (Remarks to the Author):

The manuscript of Zhang et al entitled “Arabidopsis AGDP1 links H3K9me2 to DNA methylation in heterochromatin” demonstrates that AGDP1 protein binds to H3K9me2 concluding that this interaction is essential in heterochromatinization of transposons. The authors discovered a new regulator of chromatin structure unveiling the mechanistic link between histone modification and DNA methylation. The experiments are performed appropriately with adequate controls, so the conclusions are nicely supported. The manuscript is written clearly and has a good structure as well. Here are my comments:

1. Although the authors intensively explored that AGDP1 controls chromatin through SUVH4/5/6, it is still unclear how AGDP1 mechanistically affects SUVH4/5/6. It is probably beyond the scope of the manuscript, however, it would be nice to touch this issue by showing at least the expression levels of SUVH4/5/6 in agdp1 mutants. Ideally, the protein levels and the binding of SUVH4/5/6 in agdp1 mutants would be needed to fully answer this question, but considering the availability of the tagging lines for those histone methyltransferases, I would suggest that the authors discuss this issue a bit more in the discussion section.

2. The authors argued that AGDP1 influences long TEs more strongly. The length issue of TEs can be attributed to TE classes. I would suggest to perform a simple analysis on the classification of TEs that are affected by agdp1 mutation. Another possibility is the intactness of TEs (i.e. full-length or truncated) that is also relevant to the ages of TEs. Young TEs are longer and they must be targeted for silencing more strongly. Similarly, Panda et al. (Genome Biology (2016) 17:170) suggested that long and full-length/intact TEs are more readily targets of RdDM. Although it is difficult to directly relate this manuscript to Panda et al paper as they studied different pathway, the authors can at least consider this issue and cite the paper.

Reviewer #2 (Remarks to the Author):

Review for:

“Arabidopsis AGDP1 links H3K9me2 to DNA methylation in heterochromatin”

In this manuscript, Zhang et al., start by using modified histone peptides as bait to pull down histone reader candidates from nuclear protein extract. They focus on a novel protein, AGDP1, which is preferentially enriched with H3K9me1/2/3 over unmodified H3. This protein encodes three tandem

agenet domains. Each tandem domain is shown to preferentially bind H3K9me2 in vitro and they solve the crystal structure of the first (ADG12) in complex with H3K9me2 peptide. They determine the amino acids responsible for coordinating H3K9me2 binding and describe the unique peptide binding pocket. Next they perform a series of genomic analyses on an agdp1 mutant, finding that it is required for non-CG DNA methylation, H3K9me2 and transcriptional silencing at a subset of heterochromatic loci. They perform ChIPseq on an AGDP1 tagged line and make the argument that it preferentially associates with long TEs, although the evidence for this is weak, see further comments below.

Overall this is an interesting piece of work on a novel factor with the biochemical + structural data largely in congruence with the genomics. However, while the biochemistry section provides robust novel insight, the genomic portion appears somewhat rushed and frequently lacks sufficient rigour to justify the conclusions made. Detailed comments below:

Comments

The association between H3K9me2 and AGDP1 enrichment should be investigated, as that is the central theme of this paper and AGDP1 is supposed to be directly recruited by H2K9me2. For instance the correlation could be shown as a scatter plot. The authors even performed H3K9me2 ChIP in WT and agdp1, so why not sequence to determine H2K9me2 levels genome wide? Alternatively this correlation could be easily be assessed by analysing previously published H3K9me2 ChIPseq datasets.

Overall, the evidence that AGDP1 binds preferentially to long TEs is not sufficiently rigorous. In Figure 7bc the authors try to show an association between AGDP1 ChIPseq and longer TEs, however it could simply be that longer TEs are more likely to overlap with randomly distributed peaks, by virtue of them being longer. Also, how do the authors define “AGDP1 bound” is this simply any level of overlap with a peak? Perhaps only TEs with greater than 80% overap with ChIPseq peak should be considered bound, (and this should be described in the methods)? At least the authors should perform hypergeometic analyses or compare this TE length association with a set of randomly distributed peaks of the same length distribution as the AGDP1 peaks, as was conducted for the analysis in Figure 6D. Why not do a metaplot of AGDP1 enrichment over long vs short TEs?

Line 337: “We found that CHG and CHH methylation were more affected in long TEs than in short TEs in the agdp1-1 mutant (Fig. 7d)”. The evidence does not support this statement. It is true that the absolute levels of methylation loss are higher at longer TEs, however the levels of methylation in WT are also much higher at long TEs as compared to short. If long TEs are preferentially affected, then there should be a greater relative loss, but looking at the data eg for mCHH, a change from ~7.5% to ~5.5% at TEs >4kb is about the same as a change from ~4% to ~3% at TEs <0.5kb. It is this relative change that should be quantified.

Line 407 “Previous studies showed that DDM1 is also preferentially required for the DNA methylation of long TEs35, but it is unknown why DDM1 preferentially affects long TEs” – as stated in the title of ref 35 “The Arabidopsis nucleosome remodeler DDM1 allows DNA methyltransferases to access H1-containing heterochromatin” the preference comes from a requirement for DDM1 to allow CMT2 methyltransferase access to DNA that is blocked by H1 linker. A more recent paper also from the Zilberman group provide further support for this model showing that DDM1 is required for DNA methylation to occur on nucleosomal template. It would be very interesting to see if something similar would be the case for AGDP1 in order to explain the proposed preferential effect at long TEs / heterochromatin - ie a h1/agdp1 double mutant should ameliorate methylation losses in agdp1 if AGDP1 helps to maintain CMT2 access to heterochromatin.

Line 287: The authors show a ‘global’ reduction in H2K9me2 by western but do not show how the global distribution of H3K9me2 is affected in agdp1 by ChIPseq? This should correlate with the DMR distribution and should reflect the genomic localisation of AGDP1.

Line 291: The authors show that AGDP1 is localised in the nucleus over the cytoplasm, but if AGDP1 binds to H3K9me2 and heterochromatin preferentially, it should be enriched at chromocenters within the nucleus.

Line 321: The authors already show that agdp1 generally shows reduced methylation at suvh4/5/6 dependent sites in Fig 5A so the reduction is expected in Fig 6e. It would be more convincing to show that methylation is preferentially lost at the AGDP1 bound subset of suvh4/5/6 dependent sites.

Line 393: The authors state that “AGDs of AGDP1 can specifically recognize the methylated K9 and unmethylated K4” but this is based on the crystal structure bound to a unmodified K4. It would be good to show that the dual mark H3K4me1/2/3 + H3K9me2 prevents binding by ITC.

In Figure 1b the authors show that each tandem AGD binds H3K9me2, but what about the full length? Is this protein stable? It would be interesting to determine whether the full length has additive or synergistic binding to H3K9me2. On a related note, how do the authors envisage that having multiple H3K9me2 binding sites per protein is useful in vivo? Would it simply make it more likely to bind an H3K9me2? Would it bring many nucleosomes together to coordinate packing? Presumably a single AGDP1 protein would likely only be able to bind a single nucleosome at a time?

The RNAseq and WGBS experimental setup is not explained, how many biological replicates are used per sample?

Line 216: Why were 8 TEs chosen for validation out of 15? Seems to be a bit arbitrary.

Line 243: and many places throughout the manuscript “The downregulation of DNA methylation” should be “The reduction” as methylation is likely not being downregulated in the transcriptional sense.

Line 248 “indicating that the loss of DNA methylation in agdp1-1 led to derepression of TEs.” Or visa versa could be the case.

How do the authors generate random peaks with same length distribution as shown in Fig 6D? Custom script? Should be described in the methods.

Line 402: “despite intensive efforts” – what efforts? Did the authors perform IP-MS? BiFC? Y2H screen? The authors should either present the data or remove this line from the discussion as it is ambiguous.

For browser shots as in Fig 6B no scale is shown. How are the ChIP tracks normalised? More description is required in the methods or legends. Typically enrichment is shown over WT. Similarly, in Sup Fig 9b clearly the AGDP1 tracks are off the scale so need to re-scale and to show the scale for each track. Enrichment at this region in the WT tracks indicates that this is a repetitive region.

For Figure 6F: Related to the first comment, it would be nice to show a browser track with H3K9me2 ChIPseq levels as this is what AGDP1 is supposed to directly bind. Authors would ideally use their own (they already perform H3K9me2 ChIP in WT vs agdp1-1, Fig5B, but would also need to include H3 ChIPseq for normalisation) or alternatively could use previously published datasets.

For the RNAseq and WGBS the authors used the agdp1-1 and not agdp1-2 t-dna line. Why use one and not the other? Is one agdp1-2 a hypomorph? Is there any characterisation of these t-dnas? If both are predicted to be null then one could use the overlap between the two t-dnas to more robustly define the DEGs and DMRs.

Figure 3a – RsAGDP1 AGD1 should be AGD12

Figure 4a – both venn diagrams say ‘Up’

Nothing has been deposited to GEO and a reviewer access link should be provided.

Reviewer #3 (Remarks to the Author):

The manuscript entitled "Arabidopsis AGDP1 links H3K9me2 to DNA methylation in heterochromatin" by Zhang et al. reports the crystal structure of the first pair of tandem Agenet domains of Raphanus sativus AGDP1 in complex with an H3K9me2 peptide. The authors describe the interactions in detail and identify two pockets in AGD1 and AGD2 respectively to bind K4me0 and K9me2. The fact that the histone peptide binds the tandem Agenet domains in a helical conformation is clearly different from previously reported SAWADEE-H3K9me2 complex, which is an is an important observation. The authors then carry out a number of in vivo assays, i.e. ChIP-seq and RNA-seq, to explore the function of AGDP1. AGDP1 is required for transcription silencing and methylation of CHG and CHH. Overall, the manuscript presents interesting work and brings an important contribution to the TE silencing field in plant. This study contained a lot of structural and genetic analyses and the data were mostly nicely shown. In my opinion, the manuscript is worthy of publication in Nat. Comm. with minor revision.

1. Fig. 1b-d showed the ITC results of the binding affinities of the tandem AGD domains of AtAGDP1 to H3K9 peptides. The authors then use the structure of RsAGD12 to resemble AtAGD12 due to the high sequence identity between the two species. Residues involved in the peptide binding are also conserved. However, it is noteworthy that the binding affinities of AtAGD12 for H3K9me1 and

H3K9me2 are similar, only 1.1-fold different. Can the authors interpret why AtAGD12 shows much weaker preference over H3K9me2?

2. The authors declare that AGD12 specifically recognize the H3K4me0 and H3K9me2 marks. The only evidence for the recognition of H3K4me0 is that in the crystal structure, K4unme forms hydrogen bonding and salt bridge interactions with Glu89 and Glu45. Either E89A or E45A mutant disrupts these interactions. However, these two mutants couldn't discriminate the methylation status of K4. ITC experiments should be performed using histone peptides with different methylation status on K4, e.g. K4me1.

3. p11, the authors concluded that the three pairs of the tandem AGD domains of AGDP1 share the same H3K9me2-recognition mechanisms. The conclusion was made based on the sequence alignment and the binding feature of AtAGD12, AtAGD34, AtAGD56, and RsAGD12. The inclusion of RsAGD34 and RsAGD56 in the sequence alignment as well as the ITC data of RsAGD34 and RsAGD56 binding to different H3K9me peptides would make the conclusion more persuasive.

4. Fig. 1f showed the overall structure of AGD12 is similar to FMRP tandem-AGD. An r.m.s.d. value should be provided based on structural superimposition. Fig. 1h showed that the H3 peptide adopts a helical conformation as in BAZ2A PHD-H3 complex. Again, an r.m.s.d. value should be provided. I suggest to move Fig. 1f and 1h to Supplementary section. Fig. 1g could be presented as stereo view images.

5. Fig3a, AtAGD34 adopts almost identical overall structure to RsAGD12. However, as shown in Fig 1c, AtAGD34 binds to H3K9me3 with the Kd of 189 micormolar, reflecting a 14-fold reduction in binding affinity compared with the dimethylated H3K9 peptide. In contrast, RsAGD12 only exhibits a 5-fold reduction in binding to H3K9me3 compared with H3K9me2. The authors need to discuss in-depth the mechanism of the selectivity of the methylation level by different AGDs.

6. Histone H3K27 shares a four amino acid ARKS motif with H3K9. Is it possible that AGD12 could also bind H3K27?

7. Furthermore, A1, R2 and R8 also contribute to the binding. Evidence for the disruption of the interactions between these residues and AGD12 should be provided.

8. The dimethyl group of H3K9me2 is accommodated by a classical aromatic cage, which is composed of three aromatic residues and one negatively charged residue. Other histone readers are

usually found to use the same type of aromatic cage to bind di-/trimethyllysine, i.e. the PHD domain of UHRF1 (4GY5), the chromodomain of Mpp8 (3R93). Please discuss why the cage in AGDP prefers to bind mono-/dimethylated lysine over trimethylated lysine.

Reviewer #4 (Remarks to the Author):

Comments on Zhang et al. NCOMMS-18-09908-T

In this manuscript, Zhang et al. identified a novel reader of H3K9 methylation marks, AGDP1, in Arabidopsis thaliana by a mass-spectrometry based screening approach. AGDP1 contains 6 agenet domains (AGD), which can be further grouped into three tandem AGD pairs (AGD12, AGD34 and AGD56). The authors showed that each AGD pair preferentially binds to H3K9 methylated marks. They obtained high-resolution structures of RsAGD12 and AtAGD34 with histone peptides, which nicely confirm the interaction between H3K9me2 and AGDs. The authors also found that AGDP1 is involved in genome-wide non-CG methylation and it preferentially occupies long TE regions. Overall, the quality of this manuscript is high. However, there are several major concerns to address before publication in Nature Communications:

1. The mass-spectrometry approach used in the initial screening needs to be described in detail. According to the Supplemental Experimental Procedures in the referenced article (#36; Lang, Z. et al. 2015 Mol Cell), the captured proteins were "eluted from the beads with PBS and denatured in 8M urea and reduced with 5 mM dithiothreitol for 30 min at 50 °C". How were the proteins eluted from beads by PBS (I assume it is Phosphate Buffered Saline – please clarify)? Or, were elution, denaturation and reduction done simultaneously by 8M urea and 5 mM DTT in PBS? Considering the sensitivity of MS, it is hard to conceive that the analysis detected only five proteins described in Supplemental Table 1. How were background proteins filtered out in the analyses? Does Supplemental Table 1 show ALL peptides detected in each mass spectrometry run (then why are there no histones?), or is it a snippet summarizing previously known/histone-related factors detected in the analysis? Clarification of these things will help readers understand what data are being presented. The complete mass spec datasets should also be deposited in a public repository, or made available.

2. The authors obtained high quality crystals of RsAGD12 and AtAGD34, but not crystals of full-length protein. Did they try to solve the full-length AGDP1 structure? Do they have any insights into how the three tandem AGD pairs are organized within the full-length protein?

3. The authors expressed three AGD pairs from Arabidopsis AGDP1 and showed that they all preferentially bind to methylated H3K9 marks (Fig. 1). The structures of RsAGD12 and AtAGD34 also confirm that each AGD pair can bind a H3K9me2 peptide (Fig. 1 to 3). Can the authors further show how many H3K9me2 peptides can be bound by a full-length AGDP1 protein? Given the fact that AGDP1 has three pairs of AGDs, does each AGDP1 bind to three histone tails of one nucleosome or does each AGDP1 bind to multiple histone tails from multiple nucleosomes?

4. In Line 201, the subtitle 'AGDP1 is required for transcriptional silencing' is a strong statement. Only 15 TEs and 68 genes were upregulated in agdp1-1 mutant (Fig. 4a). However, there were also a handful of TEs and dozens of genes that were downregulated in agdp1-1 (Supplemental Table 3). Since the number of up- and down-regulated TEs/genes are comparable in agdp1-1, there is not strong evidence that AGDP1 is directly required for transcriptional silencing.

5. Fig. 4a and Supplemental Table 3: Many "genes" described in these data are contaminated with transposable element genes, and therefore overlap with separately analyzed "TE" annotations. Among 68 "genes" upregulated in agdp1-1 mutant, 13 of them (AT2G16680, AT3G50625, AT3G28915, AT5G07215, AT5G27345, AT3G42658, AT3G60170, AT2G05040, AT5G19097, AT3G43828, AT4G01490, AT1G20390, AT5G34790) are classified as "transposable_element_gene" by TAIR10. Most of these genes are associated with corresponding TE annotation - for example, AT2G16680 is associated with AT2TE29460; AT3G50625 is associated with AT3TE76225; AT3G28915 is associated with AT3TE45385, and so on. Likewise, 258 of 632 "genes" derepressed in suvh4/5/6 are actually "transposable_element_gene". Please carefully revise the annotations of "genes" and "TE", and redo the analyses..

6. Line 402-404: The possibility of AGDP1 being directly involved in structural changes of chromatin rather than recruitment of other factors should be discussed more carefully, because currently it is not clear what was tested in their "intensive efforts" to identify AGDP1-associated factors. Did they try yeast two-hybrid, immunoprecipitation or mass spectrometry? Since they successfully conducted ChIP on Flag-AGDP1 (Fig. 6), one suspects that it shouldn't be an immunoprecipitation issue. Did they try IP-mass spectrometry from nuclear extracts as used in the initial screening? Because the possibility that AGDP1's direct involvement in chromatin structural change stems from negative results (ie no AGDP1-associated proteins identified), the experiments conducted and the outcome should be explained.

7. Please clearly describe the parameters used for high-throughput sequencing data analysis, e.g. mapping parameters with TopHat2, p-value or FDR used for DE analysis.

Minor comments

(1) In Abstract, please define what “AGD12” means (line 30).

(2) Please check the manuscript thoroughly for “micro” symbols and fix “u” to “µ”. Examples are line.440, line.444 and line.534.

We thank all the reviewers for their positive and constructive comments. We have performed additional bioinformatic analyses, H3K9me2 and H3 ChIP-Seq, subnuclear localization, and ITC experiments according to the reviewers' suggestion and made our point-to-point response as below with our response highlighted in red.

Reviewers' comments:

Reviewer #1 (Remarks to the Author):

The manuscript of Zhang et al entitled "Arabidopsis AGDP1 links H3K9me2 to DNA methylation in heterochromatin" demonstrates that AGDP1 protein binds to H3K9me2 concluding that this interaction is essential in heterochromatinization of transposons. The authors discovered a new regulator of chromatin structure unveiling the mechanistic link between histone modification and DNA methylation. The experiments are performed appropriately with adequate controls, so the conclusions are nicely supported. The manuscript is written clearly and has a good structure as well. Here are my comments:

We thank the reviewer for the positive comments to our manuscript.

1. Although the authors intensively explored that AGDP1 controls chromatin through SUVH4/5/6, it is still unclear how AGDP1 mechanistically affects SUVH4/5/6. It is probably beyond the scope of the manuscript, however, it would be nice to touch this issue by showing at least the expression levels of SUVH4/5/6 in agdp1 mutants. Ideally, the protein levels and the binding of SUVH4/5/6 in agdp1 mutants would be needed to fully answer this question, but considering the availability of the tagging lines for those histone methyltransferases, I would suggest that the authors discuss this issue a bit more in the discussion section.

Our RNA-seq data showed that the expression levels of *CMT2*, *CMT3*, *SUVH4*, *SUVH5*, *SUVH6* and *DDM1* were not affected in *agdp1-1* (**Supplementary Table 3**), indicate that the reduction of non-CG methylation and H3K9me2 in *agdp1* mutants was not caused by the reduced expression of these genes. To explore the mechanism of AGDP1 function, we performed IP-MS analysis with four replicates, but did not find any interaction between AGDP1 and these enzymes (**Supplemental Table 7**). Considering that, except the three tandem AGD cassettes, there are no other functional domain was found in AGDP1 protein, it is possible that AGDP1 does not directly recruit chromatin-modifying enzymes but instead serves in a structural role. As suggested, we have discussed this issue in the discussion section.

2. The authors argued that AGDP1 influences long TEs more strongly. The length issue of TEs can be attributed to TE classes. I would suggest to perform a simple analysis on the classification of TEs that are affected by agdp1 mutation. Another possibility is the intactness of TEs (i.e. full-length or truncated) that is also relevant to the ages of TEs. Young TEs are longer and they must be targeted for silencing more strongly. Similarly, Panda et al. (Genome Biology (2016) 17:170) suggested that long and full-length/intact

TEs are more readily targets of RdDM. Although it is difficult to directly relate this manuscript to Panda et al paper as they studied different pathway, the authors can at least consider this issue and cite the paper.

As suggested, we have performed the classification of TEs that are affected by *agdp1*. We found that DNA/En-Spm, LTR/Copia, LTR/Gypsy are enriched in both CHG and CHH hypo-DMRs (**Supplemental Table 5**). In addition, compared to the truncated TEs, the full length TEs are enriched in both CHG and CHH hypo-DMRs (**Supplemental Table 5**). We have incorporate these data into our revised manuscript and have cited the paper of Panda et al.

Reviewer #2 (Remarks to the Author):

Review for:

“Arabidopsis AGDP1 links H3K9me2 to DNA methylation in heterochromatin”

In this manuscript, Zhang et al., start by using modified histone peptides as bait to pull down histone reader candidates from nuclear protein extract. They focus on a novel protein, AGDP1, which is preferentially enriched with H3K9me1/2/3 over unmodified H3. This protein encodes three tandem agenet domains. Each tandem domain is shown to preferentially bind H3K9me2 in vitro and they solve the crystal structure of the first (ADG12) in complex with H3K9me2 peptide. They determine the amino acids responsible for coordinating H3K9me2 binding and describe the unique peptide binding pocket. Next they perform a series of genomic analyses on an agdp1 mutant, finding that it is required for non-CG DNA methylation, H3K9me2 and transcriptional silencing at a subset of heterochromatic loci. They perform ChIPseq on an AGDP1 tagged line and make the argument that it preferentially associates with long TEs, although the evidence for this is weak, see further comments below.
Overall this is an interesting piece of work on a novel factor with the biochemical + structural data largely in congruence with the genomics. However, while the biochemistry section provides robust novel insight, the genomic portion appears somewhat rushed and frequently lacks sufficient rigour to justify the conclusions made. Detailed comments below:

We thank the reviewer for the positive comments to our structure observations. We have further strengthened the genomic part by performing additional experiment and the point-to-point response is below.

Comments
The association between H3K9me2 and AGDP1 enrichment should be investigated, as that is the central theme of this paper and AGDP1 is supposed to be directly recruited by H3K9me2. For instance the correlation could be shown as a scatter plot. The authors even

performed H3K9me2 ChIP in WT and agdp1, so why not sequence to determine H3K9me2 levels genome wide? Alternatively this correlation could be easily be assessed by analysing previously published H3K9me2 ChIPseq datasets.

To test the association between H3K9me2 and AGDP1 enrichment and to determine H3K9me2 levels genome wide, we performed H3K9me2 and H3 ChIP-Seq in wild type and *agdp1-1* with two biological replicates. The correlation of AGDP1 enrichment with H3K9me2 modification levels has been analyzed through scatter plot, and result showed that the correlation is 0.424, suggesting that AGDP1 enrichment is associated with H3K9me2 modification (**Supplementary Fig. 8a**). In addition, our box plot analysis showed that the H3K9me2 levels are clearly reduced at the AGDP1 binding peaks and hypo-DMRs in *agdp1-1* mutants compared with wild type (**Fig. 5c and Supplementary Fig. 8b**). We have incorporate these data into our revised manuscript.

Overall, the evidence that AGDP1 binds preferentially to long TEs is not sufficiently rigorous. In Figure 7bc the authors try to show an association between AGDP1 ChIPseq and longer TEs, however it could simply be that longer TEs are more likely to overlap with randomly distributed peaks, by virtue of them being longer. Also, how do the authors define “AGDP1 bound” is this simply any level of overlap with a peak? Perhaps only TEs with greater than 80% overap with ChIPseq peak should be considered bound, (and this should be described in the methods)? At least the authors should perform hypergeometic analyses or compare this TE length association with a set of randomly distributed peaks of the same length distribution as the AGDP1 peaks, as was conducted for the analysis in Figure 6D. Why not do a metaplot of AGDP1 enrichment over long vs short TEs?

As suggested, only TEs with greater than 80% overap with ChIP-seq peaks were defined as “AGDP1 bound TEs” and we have described this in figure legends and redo the analysis for **Figure 7b**. To exclude the possibility that longer TEs are more likely to overlap with randomly distributed peaks, we added three sets of randomly distributed peaks with the same length distribution of the AGDP1 peaks as control for Figure 7b analysis. Through metaplot analysis of AGDP1 enrichment over long vs short TEs, we found that AGDP1 preferentially binds to TEs that are longer than 4 Kb (**Fig. 7c and Supplementary Fig.10**). We have replaced Fig. 7c with the new Metaplot result and have incorporate these data into our revised manuscript.

Line 337: “We found that CHG and CHH methylation were more affected in long TEs than in short TEs in the agdp1-1 mutant (Fig. 7d)”. The evidence does not support this statement. It is true that the absolute levels of methylation loss are higher at longer TEs, however the levels of methylation in WT are also much higher at long TEs as compared to short. If long TEs are preferentially affected, then there should be a greater relative loss, but looking at the data eg for mCHH, a change from ~7.5% to ~5.5% at TEs >4kb is about the same as a change from ~4% to ~3% at TEs <0.5kb. It is this relative change that should be quantified.

We agree with the reviewer that the relative changes of DNA methylation at longer TEs are not greater than short TEs in *agdp1* mutant. But many other researchers (Zemach, A. et al., Cell, 2013; Panda, K. et al., Genome Biol., 2016) have used the absolute levels of methylation loss for their DNA methylation change analysis with metaplot. To avoid misleading, we have modified the sentence to "We found that the absolute levels of CHG and CHH methylation loss are higher at longer TEs (**Fig. 7d**)".

Line 407 "Previous studies showed that DDM1 is also preferentially required for the DNA methylation of long TEs35, but it is unknown why DDM1 preferentially affects long TEs" – as stated in the title of ref 35 "The Arabidopsis nucleosome remodeler DDM1 allows DNA methyltransferases to access H1-containing heterochromatin" the preference comes from a requirement for DDM1 to allow CMT2 methyltransferase access to DNA that is blocked by H1 linker. A more recent paper also from the Zilberman group provide further support for this model showing that DDM1 is required for DNA methylation to occur on nucleosomal template. It would be very interesting to see if something similar would be the case for AGDP1 in order to explain the proposed preferential effect at long TEs / heterochromatin - ie a h1/agdp1 double mutant should ameliorate methylation losses in agdp1 if AGDP1 helps to maintain CMT2 access to heterochromatin.

We apologize for the confusion of our sentences. We have rewritten the sentence as the following: "Previous studies showed that DDM1 allow methyltransferase access to nucleosomal templates and longer TEs that are blocked by the H1 linker[39,40]. It is still unknown why DDM1 preferentially affects the chromatin architecture of long TEs but not short TEs." In addition, we have cited the more recent paper of Zilberman group which report that DDM1 is required for DNA methylation to occur on nucleosomal template. We also think that it would be interesting to see if histone *h1* mutation can suppress *agdp1* mutant phenotype by future studies. Currently, we are not able to get a conclusion before we obtained the *agdp1 h1.1 h1.2* triple mutant plants. We have included the sentence "It would be interesting to see if histone *h1* mutation can suppress *agdp1* mutant phenotype by future studies" in our revised manuscript.

Line 287: The authors show a 'global' reduction in H3K9me2 by western but do not show how the global distribution of H3K9me2 is affected in agdp1 by ChIPseq? This should correlate with the DMR distribution and should reflect the genomic localization of AGDP1.

To investigate the global distribution of H3K9me2, we performed H3K9me2 and H3 ChIP-Seq in WT and *agdp1-1* with two biological replicates. The results showed that the H3K9me2 levels are reduced at AGDP1-binding regions and hypo-DMRs in *agdp1-1* mutants compared with WT (**Fig. 5c and Supplementary Fig. 8b**). We have added our new data in our revised manuscript.

Line 291: The authors show that AGDP1 is localised in the nucleus over the cytoplasm,

but if AGDP1 binds to H3K9me2 and heterochromatin preferentially, it should be enriched at chromocenters within the nucleus.

We have tried to do immunostaining with *pAGDP1-Flag-AGDP1* transgenic plants to detect the co-localization of AGDP1 with H3K9me2. However, although we were capable of detecting the strong signal of H3K9me2, but failed to detect the *Flag-AGDP1* signal. To further explore the subnuclear localization of AGDP1, we infiltrated *Nicotiana benthamiana* leaves with *p35S-GFP-AGDP1*. We found that, the fluorescence of the control protein GFP, was dispersed in the nuclear and excluded from the nucleolus regions, GFP-AGDP1 fluorescence showed similar localization patterns with DAPI staining, except for the strong fluorescence signal within the nucleolus (**Supplementary Fig. 6**). We have replaced the subcellular localization of AGDP1 with the new subnuclear localization results and have added a short discussion in the revised manuscript.

Line 321: The authors already show that agdp1 generally shows reduced methylation at suvh4/5/6 dependent sites in Fig 5A so the reduction is expected in Fig 6e. It would be more convincing to show that methylation is preferentially lost at the AGDP1 bound subset of suvh4/5/6 dependent sites.

The **Fig. 5a** showed that *agdp1* generally shows reduced methylation at *suvh4/5/6* dependent sites. However, **Fig. 6e** showed that the CHG and CHH methylation levels were decreased in *agdp1-1* and *suvh4/5/6* mutants at AGDP1-binding regions. As suggested, we have analyzed the methylation levels of the AGDP1 bound subset of *suvh4/5/6* dependent sites. The new data below showed that the loss of DNA methylation at the AGDP1 bound subset of *suvh4/5/6* dependent sites were comparable to **Fig. 6e**, so we will not include this in the revised manuscript.

Fig. 6e

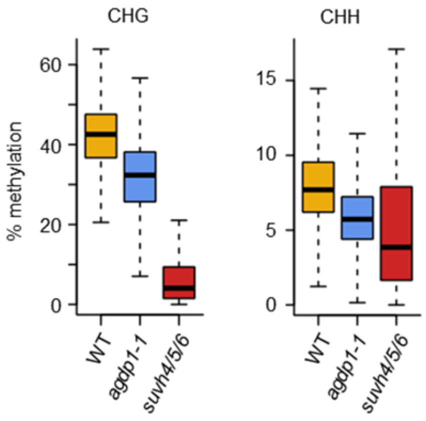

New data

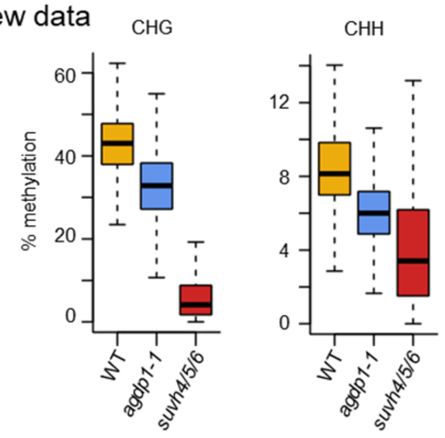

Line 393: The authors state that “AGDs of AGDP1 can specifically recognize the methylated K9 and unmethylated K4” but this is based on the crystal structure bound to a

unmodified K4. It would be good to show that the dual mark H3K4me1/2/3 + H3K9me2 prevents binding by ITC.

As suggested, we have performed ITC binding between RsAGDP1 AGD12 and H3K4me1/2/3K9me2 peptides (**Supplemental Fig. 1g**). The data showed that the H3K4me1K9me2 and the H3K4me2K9me2 slightly decrease the binding and can be tolerated, while H3K4me3K9me2 significantly decrease the binding, suggesting the increase of H3K4 methylation state leads to decrease of the peptide binding and confirming our notion that the tandem AGD cassettes prefer H3K4me0K9me2 state. We have incorporate these data into our revised manuscript.

In Figure 1b the authors show that each tandem AGD binds H3K9me2, but what about the full length? Is this protein stable? It would be interesting to determine whether the full length has additive or synergistic binding to H3K9me2. On a related note, how do the authors envisage that having multiple H3K9me2 binding sites per protein is useful in vivo? Would it simply make it more likely to bind an H3K9me2? Would it bring many nucleosomes together to coordinate packing? Presumably a single AGDP1 protein would likely only be able to bind a single nucleosome at a time?

The full length AtAGDP1 is stable and can be expressed in *E. coli*. We were not able to obtain the crystals of the full length AtAGDP1 either alone or in complex with H3K9me2 peptide, which is probably due to the flexible linker regions between the three tandem AGD cassettes. Instead, we tested the binding between full length AtAGDP1 with H3K9me1/2/3/0 peptides (**Fig. 1e**). As expected, the AtAGDP1 show similar binding preference towards H3K9me2 and the ITC binding yield a binding stoichiometry of ~3, confirming our previous speculation that a single AGDP1 protein can bind to 3 H3K9me2 tails. In this case, it is possible that AGDP1 may bring two or three nucleosomes together to coordinate packing, although this requires more experimental confirmation. We have added our new data and a short discussion about the related interpretation in our revised manuscript.

The RNAseq and WGBS experimental setup is not explained, how many biological replicates are used per sample?

We performed RNA deep sequencing assay with five replicates for wild type and *agdp1-1*, and three replicates for *suvh4/5/6*. We performed one replicate for WGBS experiment, and the reduction of DNA methylation was confirmed by chop-PCR. We have described the RNAseq and WGBS experimental setup in detail in our revised manuscript.

Line 216: Why were 8 TEs chosen for validation out of 15? Seems to be a bit arbitrary.

To test if the TE’s silencing was also affected in another allele of *agdp1* mutant (*agdp1-2*), eight upregulated TEs were selected for RT-Q-PCR analysis. Now we have performed RT-Q-PCR for the remaining 7 TEs, and found that 4 TEs were upregulated, and the other 3 TEs were not upregulated in *agdp1-1* and *agdp1-2*. We have deleted Fig.4a and

added our new RT-Q-PCR data to **Supplementary Fig. 3**. We have now rewritten this part in our revised manuscript and have changed the subtitle to "AGDP1 contribute to transcriptional silencing of a small subset TEs" in the revised manuscript.

Line 243: and many places throughout the manuscript "The downregulation of DNA methylation" should be "The reduction" as methylation is likely not being downregulated in the transcriptional sense.

We thank the reviewer for notice us this. We have changed "The downregulation of DNA methylation" to "The reduction".

Line 248 "indicating that the loss of DNA methylation in agdp1-1 led to derepression of TEs." Or visa versa could be the case.

We agree with the reviewer. We cannot exclude the possibility that the chromatin structure change or chromatin modification might led to the derepression of these TEs. Due to lack of evidence that the derepression of these TEs was directly caused by the loss of DNA methylation, we have deleted this sentence and Supplementary Fig. 4.

How do the authors generate random peaks with same length distribution as shown in Fig 6D? Custom script? Should be described in the methods.

The random peaks with the same length distribution is generated by "shuffle" command from the BEDTools suite. It has been described in the methods.

Line 402: "despite intensive efforts" – what efforts? Did the authors perform IP-MS? BiFC? Y2H screen? The authors should either present the data or remove this line from the discussion as it is ambiguous.

To identify AGDP1-associated factors, we performed IP-MS analysis with *pAGDP1-Flag-AGDP1* transgenic plants for four replicates, but did not identify SUVH4/5/6, CMT2/3, and DDM1 from the Flag-AGDP1 IP samples (**Supplemental Table 7**). We have deleted the sentence "despite intensive efforts" and added the Flag-AGDP1 IP-MS data in our revised manuscript.

For browser shots as in Fig 6B no scale is shown. How are the ChIP tracks normalized? More description is required in the methods or legends. Typically enrichment is shown over WT. Similarly, in Sup Fig 9b clearly the AGDP1 tracks are off the scale so need to re-scale and to show the scale for each track. Enrichment at this region in the WT tracks indicates that this is a repetitive region.

As suggested, AGDP1 tracks in **Supplementary Fig. 9b** have been re-scaled. All scales in **Fig. 6B**, **6F**, and **S9** have been added. For the snapshots, the normalization factor is

calculated by the total mapped reads. The y axis is normalized read counts per million (ChIP-seq) or ten million (RNA-seq). We have described the normalization method in figure legends.

For Figure 6F: Related to the first comment, it would be nice to show a browser track with H3K9me2 ChIPseq levels as this is what AGDP1 is supposed to directly bind. Authors would ideally use their own (they already perform H3K9me2 ChIP in WT vs agdp1-1, Fig5B, but would also need to include H3 ChIPseq for normalisation) or alternatively could use previously published datasets.

We have performed H3K9me2 and H3 ChIP-Seq in WT and *agdp1-1* with two biological replicates. The browser tracks of H3K9me2 ChIPseq levels has been added in **Fig. 6F** and **Supplementary Fig. 9**.

For the RNAseq and WGBS the authors used the agdp1-1 and not agdp1-2 t-dna line. Why use one and not the other? Is one agdp1-2 a hypomorph? Is there any characterisation of these t-dnas? If both are predicted to be null then one could use the overlap between the two t-dnas to more robustly define the DEGs and DMRs.

We have added the genotyping results of *agdp1-1* and *agdp1-2* T-DNA lines in **Supplementary Fig. 3b**. In *agdp1-1*, the T-DNA was inserted into exon 1 (**Supplementary Fig. 3a**); however, in *agdp1-2*, the T-DNA was inserted into the end region of exon 3 (**Supplementary Fig. 3a**), which is closed to the stop codon of AGDP1. Our RT-PCR results of **Fig. 4a**, **4b**, and **Supplementary Fig. 3d** showed that the expression level of 10 TEs in *agdp1-2* are lower than that in *agdp1-1*, suggesting *agdp1-2* may be a hypomorph. Consistent with the weak phenotype of *agdp1-2*, RT-PCR analysis revealed a partial 1402bp transcript (the full length CDS of *AGDP1* is 1554bp) was detected in *agdp1-2* (**Supplementary Fig. 3c**). Because we cannot exclude the possibility that *agdp1-2* is a hypomorph, we only use *agdp1-1* for RNA-seq and WGBS analysis.

Figure 3a – RsAGDP1 AGD1 should be AGD12

In **Fig. 3a**, we labelled the AGD1 and AGD2 of RsAGDP1 AGD12 separately and colored them to magenta and green, respectively.

Figure 4a – both venn diagrams say ‘Up’

We have deleted Fig.4a and added our new RT-Q-PCR data to **Supplementary Fig. 3d**.

Nothing has been deposited to GEO and a reviewer access link should be provided.

All high-throughput sequencing data generated in this study have been deposited in GEO with accession codes GSE111609 [Go to https://www.ncbi.nlm.nih.gov/geo/query/acc.cgi?acc=GSE111609, Enter token

qjcdoyeofnsnpub into the box]. X-ray structures have been deposited in the RCSB Protein Data Bank with accession codes 5ZWX [http://www.rcsb.org/structure/5zwx] for RsAGDP1 AGD12-H3K9me2 complex and 5ZWZ [http://www.rcsb.org/structure/5zwz] for AtAGDP34.

Reviewer #3 (Remarks to the Author):

The manuscript entitled "Arabidopsis AGDP1 links H3K9me2 to DNA methylation in heterochromatin" by Zhang et al. reports the crystal structure of the first pair of tandem Agenet domains of Raphanus sativus AGDP1 in complex with an H3K9me2 peptide. The authors describe the interactions in detail and identify two pockets in AGD1 and AGD2 respectively to bind K4me0 and K9me2. The fact that the histone peptide binds the tandem Agenet domains in a helical conformation is clearly different from previously reported SAWADEE-H3K9me2 complex, which is an is an important observation. The authors then carry out a number of in vivo assays, i.e. ChIP-seq and RNA-seq, to explore the function of AGDP1. AGDP1 is required for transcription silencing and methylation of CHG and CHH. Overall, the manuscript presents interesting work and brings an important contribution to the TE silencing field in plant. This study contained a lot of structural and genetic analyses and the data were mostly nicely shown. In my opinion, the manuscript is worthy of publication in Nat. Comm. with minor revision.

We thank the reviewer for the positive comments to our manuscript.

1. Fig. 1b-d showed the ITC results of the binding affinities of the tandem AGD domains of AtAGDP1 to H3K9 peptides. The authors then use the structure of RsAGD12 to resemble AtAGD12 due to the high sequence identity between the two species. Residues involved in the peptide binding are also conserved. However, it is noteworthy that the binding affinities of AtAGD12 for H3K9me1 and H3K9me2 are similar, only 1.1-fold different. Can the authors interpret why AtAGD12 shows much weaker preference over H3K9me2?

We have generate an AtAGD12 structure model based on the RsAGDP1 AGD12 structure using SWISS-MODEL. However, we failed to identify any difference in the H3K9me2 binding pocket, as the pocket is quite conserved (**Fig. 3b**). We think that some minor differences outside the H3K9me2 binding pocket may slightly change the shape and charge of the H3K9me2 binding pockets, leading to different sub-level preference of different tandem AGD cassettes. Currently, we are not able to explain them just by the currently available structure information. We have included a short discussion about this point in text of our revised manuscript.

2. The authors declare that AGD12 specifically recognize the H3K4me0 and H3K9me2 marks. The only evidence for the recognition of H3K4me0 is that in the crystal structure, K4unme forms hydrogen bonding and salt bridge interactions with Glu89 and Glu45. Either E89A or E45A mutant disrupts these interactions. However, these two mutants

couldn't discriminate the methylation status of K4. ITC experiments should be performed using histone peptides with different methylation status on K4, e.g. K4me1.

As suggested, we have performed ITC binding between AGD12 and H3K4me1/2/3K9me2 peptides (new **Supplemental Fig. 1g**). The data showed that the H3K4me1K9me2 and H3K4me2K9me2 slightly decrease the binding and can be tolerated, while the H3K4me3K9me2 significantly decrease the binding, suggesting the increase of H3K4 methylation state leads to decrease of the peptide binding. We have incorporate these data into our revised manuscript.

3. p11, the authors concluded that the three pairs of the tandem AGD domains of AGDP1 share the same H3K9me2-recognition mechanisms. The conclusion was made based on the sequence alignment and the binding feature of AtAGD12, AtAGD34, AtAGD56, and RsAGD12. The inclusion of RsAGD34 and RsAGD56 in the sequence alignment as well as the ITC data of RsAGD34 and RsAGD56 binding to different H3K9me peptides would make the conclusion more persuasive.

We have performed ITC binding between RsAGDP1 AGD34, AGD56 and different H3K9 methylated peptide and all of them show similar binding preference to H3K9me2 (**Supplementary Fig. 1b,c**). We also include them in the sequence alignment of the new **Fig. 3b**, too. The new data are incorporated into our revised manuscript.

4. Fig. 1f showed the overall structure of AGD12 is similar to FMRP tandem-AGD. An r.m.s.d. value should be provided based on structural superimposition. Fig. 1h showed that the H3 peptide adopts a helical conformation as in BAZ2A PHD-H3 complex. Again, an r.m.s.d. value should be provided. I suggest to move Fig. 1f and 1h to Supplementary section. Fig. 1g could be presented as stereo view images.

The superimposition of AGD12 and FMRP tandem-AGD yield an RMSD of 1.9 Å and the superimposition of H3K9me2 peptide and BAZ2A PHD-H3 yield an RMSD of 0.6 Å. We have added these information into our revised draft. We have also revised our figures according to the reviewer's suggestion.

5. Fig3a, AtAGD34 adopts almost identical overall structure to RsAGD12. However, as shown in Fig 1c, AtAGD34 binds to H3K9me3 with the Kd of 189 micormolar, reflecting a 14-fold reduction in binding affinity compared with the dimethylated H3K9 peptide. In contrast, RsAGD12 only exhibits a 5-fold reduction in binding to H3K9me3 compared with H3K9me2. The authors need to discuss in-depth the mechanism of the selectivity of the methylation level by different AGDs.

Because the AGD34 is an apo structure without the bounded peptide and there will be some conformational change upon peptide binding, we cannot get a clear conclusion about why the different AGD cassettes show different preferences on the H3K9me1/2/3 peptides before we obtained all the structures of AGD12-H3K9me2, AGD34-H3K9me2, AGD56-H3K9me2 complexes. As in the response to point 1, we have modelled AGD12 and AGD56 based on the current structure and sequence alignment but see no substantial

differences in the H3K9me2 binding pocket. We think that some minor differences outside the H3K9me2 binding pocket may slightly change the shape and charge of the H3K9me2 binding pockets, leading to different sub-level preference of different tandem AGD cassettes. Currently, we are not able to explain them just by the currently available structure information. We can only explain and we want to emphasize the mechanism of the H3K9me2 preference which is significant. In combination with the response to point 1, we have added a short discussion about the differences in the sub-level preferences of the different tandem AGD cassettes.

6. Histone H3K27 shares a four amino acid ARKS motif with H3K9. Is it possible that AGD12 could also bind H3K27?

As the n-5 position of H3K9me2 is the H3K4 which is also specifically recognized by AGDP1 tandem AGDs, the n-5 position of H3K27 is the H3T22 which cannot form the same recognition. We thought AGD12 is unlikely to recognize H3K27me2, which is confirmed by our ITC data below. Since we have strengthened the H3K4me0 specific recognition of AGD12, we will not include the H3K27me2 binding data in the revised manuscript.

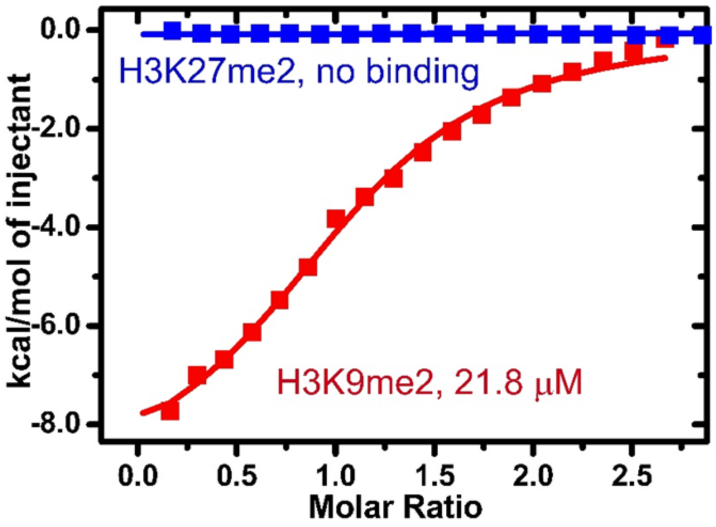

7. Furthermore, A1, R2 and R8 also contribute to the binding. Evidence for the disruption of the interactions between these residues and AGD12 should be provided.

The H3A1 is recognized by the main chain-main chain hydrogen bonds, which is hard to design mutations to abolish the binding while keeping the overall fold of the protein. So, we did not design mutation to disrupt the H3A1 binding. The mutation of Asp124 of RsAGDP1, which forms hydrogen bonding and salt bridge interaction with H3R2, also leads to dramatically decrease of the binding affinity towards H3K9me2 peptide. The H3R8 is recognized by Glu45 of RsAGDP1, which also contributes to the recognition of

H3K4. The E45A mutation also dramatically decreased the peptide binding. We have included our new data in the revised manuscript in the new **Fig. 2f**.

8. The dimethyl group of H3K9me2 is accommodated by a classical aromatic cage, which is composed of three aromatic residues and one negatively charged residue. Other histone readers are usually found to use the same type of aromatic cage to bind di-/trimethyllysine, i.e. the PHD domain of UHRF1 (4GY5), the chromodomain of Mpp8 (3R93). Please discuss why the cage in AGDP prefers to bind mono-/dimethylated lysine over trimethylated lysine.

We thank the reviewer for noticing us this point. We have made a comparison between the trimethyllysine preferred aromatic cages (MPP8 and UHRF1) and the lower methyllysine preferred aromatic cages (plant AGDP1 and mouse ORC1) (**Fig. 3c-f**). We found the former ones are relatively open and the negatively charged residues locate aside of the aromatic cage, which allows the higher methyllysine fit in. In contrast, the later ones are relatively closed with the negatively charged residues locating the opposite side of the aromatic cage to form direct hydrogen bonds with the free proton of the lower methyllysine, explaining the molecular mechanism of their preferences. We have add a short discussion and supplement the related figures in our revised manuscript.

Reviewer #4 (Remarks to the Author):

Comments on Zhang et al. NCOMMS-18-09908-T

In this manuscript, Zhang et al. identified a novel reader of H3K9 methylation marks, AGDP1, in Arabidopsis thaliana by a mass-spectrometry based screening approach. AGDP1 contains 6 agenet domains (AGD), which can be further grouped into three tandem AGD pairs (AGD12, AGD34 and AGD56). The authors showed that each AGD pair preferentially binds to H3K9 methylated marks. They obtained high-resolution structures of RsAGD12 and AtAGD34 with histone peptides, which nicely confirm the interaction between H3K9me2 and AGDs. The authors also found that AGDP1 is involved in genome-wide non-CG methylation and it preferentially occupies long TE regions. Overall, the quality of this manuscript is high. However, there are several major concerns to address before publication in Nature Communications:

We thank the reviewer for general positive comments to our manuscript.

1. The mass-spectrometry approach used in the initial screening needs to be described in detail. According to the Supplemental Experimental Procedures in the referenced article (#36; Lang, Z. et al. 2015 Mol Cell), the captured proteins were “eluted from the beads with PBS and denatured in 8M urea and reduced with 5 mM dithiothreitol for 30 min at 50 °C”. How were the proteins eluted from beads by PBS (I assume it is Phosphate Buffered Saline – please clarify)? Or, were elution, denaturation and reduction done

simultaneously by 8M urea and 5 mM DTT in PBS? Considering the sensitivity of MS, it is hard to conceive that the analysis detected only five proteins described in Supplemental Table 1. How were background proteins filtered out in the analyses? Does Supplemental Table 1 show ALL peptides detected in each mass spectrometry run (then why are there no histones?), or is it a snippet summarizing previously known/histone-related factors detected in the analysis?
Clarification of these things will help readers understand what data are being presented. The complete mass spec datasets should also be deposited in a public repository, or made available.

We thank the reviewer for notice us this. We have described the LC-MS/MS analysis in detail in the methods section. To identify Histone readers, the peptides captured proteins were analyzed by mass spectrometry and each mass spectrometry run detected more than six hundred proteins which include Histone H4, H2B and H2A (**Supplemental Table 1**). Five proteins described in Supplemental Table 1 was a snippet summarizing previously known/histone-related factors detected in the analysis. As suggested, we have added the complete mass spec datasets to the **Supplemental Table 1**.

2. The authors obtained high quality crystals of RsAGD12 and AtAGD34, but not crystals of full-length protein. Did they try to solve the full-length AGDP1 structure? Do they have any insights into how the three tandem AGD pairs are organized within the full-length protein?

We have extensively tried with full length AGDP1 in different construct and different combinations with histone peptide. However, we failed to obtain any hits for the crystallization. The difficulty is probably due to the flexible linker regions between the tandem AGD cassettes. Therefore, we propose that the three tandem AGD cassette are just simply linked together to bind to H3K9me2 enriched region and possibly also compacting the nucleosomes. As an alternative, we performed the ITC binding between full length AGDP1 and the methylated H3K9 peptides (new **Fig. 1e**). The details can be found in the response to point 3.

3. The authors expressed three AGD pairs from Arabidopsis AGDP1 and showed that they all preferentially bind to methylated H3K9 marks (Fig. 1). The structures of RsAGD12 and AtAGD34 also confirm that each AGD pair can bind a H3K9me2 peptide (Fig. 1 to 3). Can the authors further show how many H3K9me2 peptides can be bound by a full-length AGDP1 protein? Given the fact that AGDP1 has three pairs of AGDs, does each AGDP1 bind to three histone tails of one nucleosome or does each AGDP1 bind to multiple histone tails from multiple nucleosomes?

We have done ITC works with the full length AGDP1 which yields a binding stoichiometry of ~3 (**Fig. 1e**), confirming that each AGDP1 can bind to multiple histone tails from multiple nucleosomes. We have added our new data and corresponding discussion in our revised manuscript.

4. In Line 201, the subtitle ‘AGDP1 is required for transcriptional silencing’ is a strong statement. Only 15 TEs and 68 genes were upregulated in agdp1-1 mutant (Fig. 4a). However, there were also a handful of TEs and dozens of genes that were downregulated in agdp1-1 (Supplemental Table 3). Since the number of up- and down-regulated TEs/genes are comparable in agdp1-1, there is not strong evidence that AGDP1 is directly required for transcriptional silencing.

We agree with the reviewer that the effect of AGDP1 on transcriptional silencing is weak and have now changed the subtitle to “AGDP1 contribute to transcriptional silencing at some loci” in the revised manuscript.

5. Fig. 4a and Supplemental Table 3: Many “genes” described in these data are contaminated with transposable element genes, and therefore overlap with separately analyzed “TE” annotations. Among 68 “genes” upregulated in agdp1-1 mutant, 13 of them (AT2G16680, AT3G50625, AT3G28915, AT5G07215, AT5G27345, AT3G42658, AT3G60170, AT2G05040, AT5G19097, AT3G43828, AT4G01490, AT1G20390, AT5G34790) are classified as “transposable_element_gene” by TAIR10. Most of these genes are associated with corresponding TE annotation - for example, AT2G16680 is associated with AT2TE29460; AT3G50625 is associated with AT3TE76225; AT3G28915 is associated with AT3TE45385, and so on. Likewise, 258 of 632 “genes” derepressed in suvh4/5/6 are actually “transposable_element_gene”. Please carefully revise the annotations of “genes” and “TE”, and redo the analyses.

We thank the reviewer for notice us this. We have redo analysis for the differentially expressed protein coding genes and have added the new data to the **Supplemental Table 3**.

6. Line 402-404: The possibility of AGDP1 being directly involved in structural changes of chromatin rather than recruitment of other factors should be discussed more carefully, because currently it is not clear what was tested in their “intensive efforts” to identify AGDP1-associated factors. Did they try yeast two-hybrid, immunoprecipitation or mass spectrometry? Since they successfully conducted ChIP on Flag-AGDP1 (Fig. 6), one suspects that it shouldn't be an immunoprecipitation issue. Did they try IP-mass spectrometry from nuclear extracts as used in the initial screening? Because the possibility that AGDP1’s direct involvement in chromatin structural change stems from negative results (ie no AGDP1-associated proteins identified), the experiments conducted and the outcome should be explained.

To identify AGDP1-associated factors, we performed IP-MS analysis with *pAGDP1-Flag-AGDP1* transgenic plants for four replicates, but did not identify SUVH4/5/6, CMT2/3, and DDM1 from the Flag-AGDP1 IP samples (**Supplemental Table 7**). Considering that, except the three tandem AGD cassettes, there are no other functional domain was found in AGDP1 protein, it is possible that AGDP1 does not directly recruit chromatin-modifying enzymes but instead serves in a structural role.

7. Please clearly describe the parameters used for high-throughput sequencing data analysis, e.g. mapping parameters with TopHat2, p-value or FDR used for DE analysis.

As suggested, we have described the parameters used for high-throughput sequencing data analysis in detail in the methods section.

Minor comments

(1) In Abstract, please define what “AGD12” means (line 30).

We thank the reviewer for notice us this. We have added it.

(2) Please check the manuscript thoroughly for “micro” symbols and fix “u” to “μ”. Examples are line.440, line.444 and line.534.

We have fixed them.

Reviewer #1 (Remarks to the Author):

The authors extensively revised the manuscript and responded to all of the concerns raised. Thus, I do not have any objection to the manuscript to be published in nature communication.

Reviewer #2 (Remarks to the Author):

The authors have addressed my concerns.

Reviewer #3 (Remarks to the Author):

The authors have done a very good job in revising the paper. All my comments and concerns were answered or addressed experimentally. The paper is significantly improved compared to the earlier version. I fully support publication of this manuscript in its current form.

Reviewer #4 (Remarks to the Author):

In this revision, the authors included some new experiments, updated data analyses and clarified methods. Overall, they addressed most major concerns, but a few minor concerns remain.

- The authors performed an ITC experiment using full-length AtAGDP1 and H3K9 peptides, concluding that full-length AGDP1 prefers H3K9me2 and has a binding stoichiometry of three. The authors need to explain how they get this number of 3? Can the authors include any other experiment to confirm the stoichiometry, such as gel filtration chromatography of AGDP1 and the peptides? What is the biological significance- binding to multiple nucleosomes? Also, Kd values for the full-length AGDP1 are overall higher than for individual domains. Theoretically, one expectation would be higher affinity to substrates (ie lower Kd) due to multiple-binding sites per molecule for the full-length protein. Why would the full-length protein show lower affinity (higher Kd) for histone peptides? Perhaps interference among AGD12, 34 and 56 in the full-length protein?

- With the new DE data, effects of agdp1 on expression (either TE or protein-coding genes) still appear very limited, even with p-values, not q-values. Throughout the manuscript, the authors should tone down claims concerning the function of AGDP1 at a transcriptional level. It only "contributes to transcriptional silencing of a small subset TEs" (as described in a response to Reviewer 2), but there are much stronger statements in the text, with examples including (but not limited to):

- l.35: (Abstract) In addition, we find that AGDP1 is required for transcriptional silencing, non-CG DNA methylation, and H3K9 dimethylation.

- l.90: We also find that AGDP1 is required for transcriptional silencing, CHG and CHH methylation, and H3K9 dimethylation.

- l.262: AGDP1 contributes to transcriptional silencing at some loci (revised)

- l.455: We also demonstrated that AGDP1 is associated with heterochromatin and is required for CHG and CHH methylation, H3K9 dimethylation, and transcriptional silencing.

- l.777: Fig. 4: AGDP1 contributes to transcriptional silencing and non-CG methylation.

- Why do the authors show qPCR results for 8 out of 15 TEs in Fig. 4a and the remaining 7 in Fig. S3? It would make more sense to show the results for all TEs in just one figure. A table would also be acceptable.

- In Fig. 5c, can the authors also show the levels of H3K9me2 relative to H3 in agdp1 for CG hypo-DMRs as a control? Presumably, if AGDP1 mainly affects SUVH4/5/6-dependent CHG and CHH and H3K9me2 sites, we would expect to see less enrichment of H3K9me2 at CG hypo-DMRs.

- The authors mentioned that two replicates were analyzed for ChIP-seq experiments (Line 651). So, in Fig. 6d and 7c, the authors could show two metaplots for each biological replicate instead of just one merged curve (e.g. the red curve in Fig. 6d). That will tell readers the deviation of replicates.

- The authors now describe the MS analysis of FLAG-AGDP1 IP fraction (l.460-463; Supplemental Table 7), which clarifies the basis of a proposal for a potential structural role for AGDP1.

l.463-464, the statement: “it is possible that AGDP1 does not directly recruit chromatin-modifying enzymes" is too strong. It should be "... AGDP1 does not directly recruit SUVH4/5/6, CMT2/3 or DDM1". Not identifying SUVH4/5/6, CMT2/3 or DDM1 does not exclude the possibility of other characterized or uncharacterized chromatin modifying enzymes are recruited by AGDP1. For example, several histone deacetylases are identified in multiple replicates (Supplemental Table 7). Also, Replication Factor C1 (RFC1), previously shown to be involved in TGS and H3K9me2 levels (Liu Q., et al. 2010, Plant Cell; doi: 10.1105/tpc.110.076349), are found in three replicates.

Can the authors please elaborate on what they mean by a potential “structural role”.

- The authors have added a brief description of TopHat2 and Cufflinks analyses, but some details are still missing. For example, in Cufflinks, do the authors use a simple p-value, instead of a q-value (false-discovery-rate-adjusted p-value) for each DE evaluation described in Supplemental Table 3? If available, please include q-values in Supplemental Table 3.

- Please clarify the annotation definition used in the WGBS analysis; "gene" and "TEs" in Fig.4e; "protein-coding genes" in Fig.4f. Is it the same as DE analysis (ie Cufflinks) which have been updated in the revision? Also, in Fig.4f, labels on x-axis of upper plots are missing.

- In Supplemental Table 3, please clarify what column C and D represent. Are they FPKM values?

- l.387 and Fig. 6f, the browser annotation and genome positions are not readable, due to low resolution. Please make them readable, or clarify the genome location and the annotations in the legend (rather than just "a representative region").

We thank the reviewers for their positive comments. We have revised our manuscript as suggested and made our response as below with the responses highlighted in red.

REVIEWERS' COMMENTS:

Reviewer #1 (Remarks to the Author):

The authors extensively revised the manuscript and responded to all of the concerns raised. Thus, I do not have any objection to the manuscript to be published in nature communication.

We thank this reviewer's positive comments.

Reviewer #2 (Remarks to the Author):

The authors have addressed my concerns.

We thank this reviewer's positive comments.

Reviewer #3 (Remarks to the Author):

The authors have done a very good job in revising the paper. All my comments and concerns were answered or addressed experimentally. The paper is significantly improved compared to the earlier version. I fully support publication of this manuscript in its current form.

We thank this reviewer's positive comments.

Reviewer #4 (Remarks to the Author):

In this revision, the authors included some new experiments, updated data analyses and clarified methods. Overall, they addressed most major concerns, but a few minor concerns remain.

We thank this reviewer's overall positive comments.

● The authors performed an ITC experiment using full-length AtAGDP1 and H3K9 peptides, concluding that full-length AGDP1 prefers H3K9me2 and has a binding stoichiometry of three. The authors need to explain how they get this number of 3? Can the authors include any other experiment to confirm the stoichiometry, such as gel filtration chromatography of AGDP1 and the peptides?

What is the biological significance- binding to multiple nucleosomes? Also, Kd values for the full-length AGDP1 are overall higher than for individual domains. Theoretically, one expectation would be higher affinity to substrates (ie lower Kd) due to multiple-binding sites per molecule for the full-length protein. Why would the full-length protein show lower affinity (higher Kd) for histone peptides? Perhaps interference among AGD12, 34 and 56 in the full-length protein?

The binding stoichiometry of three was directly obtained by fitting the binding curve. As shown in the following figure:

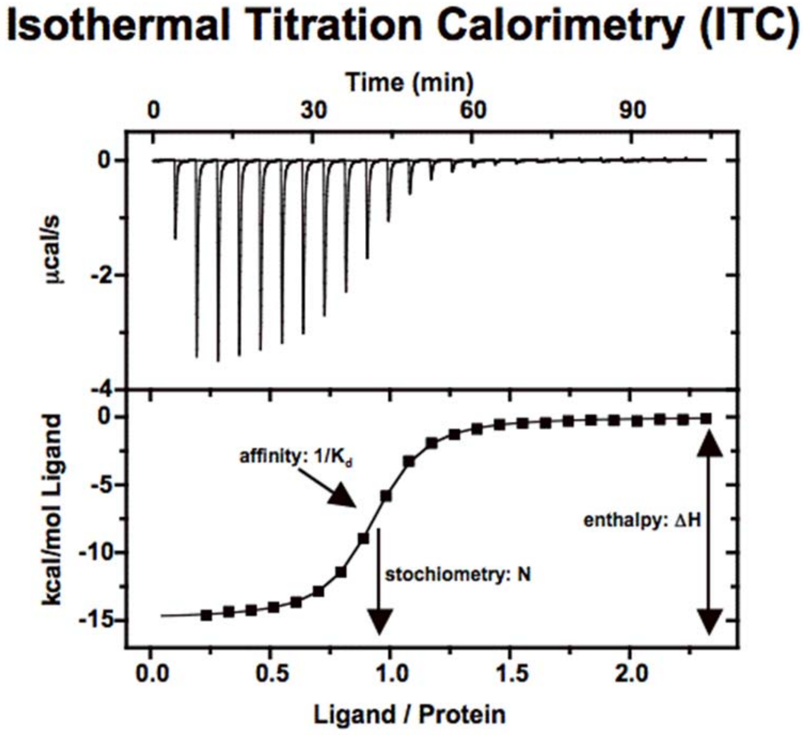

The X-axis point corresponding to the half-height of the binding curve indicates the binding stoichiometry. In our case, the half-height of the binding curves for the full length AGDP1 and H3K9me2 correspond to the binding stoichiometry. As the protein and peptide are accurately measured, we believe the ITC is best way to detect the binding stoichiometry in our case. The peptide has a quite small molecular weight. So, the binding of the peptide to AGDP1 may not show significant change to the migration rate in gel filtration. Therefore, we think gel filtration cannot be used to confirm the binding stoichiometry here.

In Line 444, we have a short discussion about the binding of AGDP1 to multiple nucleosomes that “This feature can enhance the binding of AGDP1 to the H3K9me2-enriched heterochromatin region, providing the biochemical basis for our observation of the enrichment of AGDP1 in the H3K9me2-associated long TEs.”

Because the three tandem AGDs are linked by flexible linker, we think they may bind to H3K9me2 independently and the binding affinity between the full length AGDP1 and H3K9me2 may represent the average value for the three independent bindings. However, the full length AGDP1 binds to H3K9me2 slightly weaker than the three individual domains. As indicated by the reviewer, the single global domain can freely access the histone peptide in solution. While in tandem linked conformation, there might be some interference among AGD12, 34 and 56. Some certain relative conformation of the AGD34 and AGD56 may prevent the binding of histone peptide by AGD12 transiently and *vice versa*, which may show a slightly decreased apparent affinity. As we do not have any evidence about this possibility, we will not discuss this in our manuscript.

● With the new DE data, effects of agdp1 on expression (either TE or protein-coding genes) still appear very limited, even with p-values, not q-values. Throughout the manuscript, the authors should tone down claims concerning the function of AGDP1 at a transcriptional level. It only "contributes to transcriptional silencing of a small subset TEs" (as described in a response to Reviewer 2), but there are much stronger statements in the text, with examples including (but not limited to):

● l.35: (Abstract) In addition, we find that AGDP1 is required for transcriptional silencing, non-CG DNA methylation, and H3K9 dimethylation.
● l.90: We also find that AGDP1 is required for transcriptional silencing, CHG and CHH methylation, and H3K9 dimethylation.
● l.262: AGDP1 contributes to transcriptional silencing at some loci (revised)
● l.455: We also demonstrated that AGDP1 is associated with heterochromatin and is required for CHG and CHH methylation, H3K9 dimethylation, and transcriptional silencing.
● l.777: Fig. 4: AGDP1 contributes to transcriptional silencing and non-CG methylation.

We thank the reviewer for notice us this. We have now rewritten the effects of *agdp1* on transcriptional silencing throughout the manuscript. All changes in the manuscript text have been highlighted in red.

● Why do the authors show qPCR results for 8 out of 15 TEs in Fig. 4a and the remaining 7 in Fig. S3? It would make more sense to show the results for all TEs in just one figure. A table would also be acceptable.

As suggested, we have showed the RT-qPCR results of all 15 TEs in **Fig. 4a**.

● In Fig. 5c, can the authors also show the levels of H3K9me2 relative to H3 in agdp1 for CG hypo-DMRs as a control? Presumably, if AGDP1 mainly affects SUVH4/5/6-dependent CHG and CHH and H3K9me2 sites, we would expect to see less enrichment of H3K9me2 at CG hypo-DMRs.

We have added the levels of H3K9me2 relative to H3 at CG hypo-DMRs in **Fig. 5c**. The results showed that the enrichment of H3K9me2 at CG hypo-DMRs are less than that at CHG and CHH hypo-DMRs.

● The authors mentioned that two replicates were analyzed for ChIP-seq experiments (Line 651). So, in Fig. 6d and 7c, the authors could show two metaplots for each biological replicate instead of just one merged curve (e.g. the red curve in Fig. 6d). That will tell readers the deviation of replicates.

As suggested, we have showed the metaplots for each biological replicate of Flag-AGDP1 ChIP-seq in **Fig. 7c**. The results showed that the Falg-AGDP1 ChIP-seq experiments replicate very well.

To determine which histone marks are associated with AGDP1-binding peaks, we compared the AGDP1 occupancy profiles with published genome-wide histone modification data. The **Fig. 6d** showed the levels of different histone modifications in WT at 1,914 AGDP1-enrichment peaks. The red curves in **Fig. 6d** were generated with the published genome-wide histone modification data. So, in **Fig. 6d**, only one metaplot was showed.

● The authors now describe the MS analysis of FLAG-AGDP1 IP fraction (l.460-463; Supplemental Table 7), which clarifies the basis of a proposal for a potential structural role for AGDP1.

We thank the reviewer's positive comments.

l.463-464, the statement: "it is possible that AGDP1 does not directly recruit chromatin-modifying enzymes" is too strong. It should be "… AGDP1 does not directly recruit SUVH4/5/6, CMT2/3 or DDM1". Not identifying SUVH4/5/6, CMT2/3 or DDM1 does not exclude the possibility of other characterized or uncharacterized chromatin modifying enzymes are recruited by AGDP1. For example, several histone deacetylases are identified in multiple replicates (Supplemental Table 7). Also, Replication Factor C1 (RFC1), previously shown to be involved in TGS and H3K9me2 levels (Liu Q., et al. 2010, Plant Cell; doi: 10.1105/tpc.110.076349), are found in three replicates.

We thank the reviewer for notice us this. We have changed the statement: "it is possible that AGDP1 does not directly recruit chromatin-modifying enzymes" to "… AGDP1 does not directly recruit SUVH4/5/6, CMT2/3 or DDM1" as the reviewer suggested.

Can the authors please elaborate on what they mean by a potential "structural role".

We have detailed it as 'structural role, such as acting as a scaffold protein to bridge multiple nucleosomes together.'

• The authors have added a brief description of TopHat2 and Cufflinks analyses, but some details are still missing. For example, in Cufflinks, do the authors use a simple p-value, instead of a q-value (false-discovery-rate-adjusted p-value) for each DE evaluation described in Supplemental Table 3? If available, please include q-values in Supplemental Table 3.

All the differentially expressed genes and TEs in **Supplementary Data 2** (old name: Supplemental Table 3) were identified using cuffdiff ($P < 0.05$ and log2(fold-change)>1). As suggested, we have now added q-values in **Supplementary Data 2** (old name: Supplemental Table 3).

• Please clarify the annotation definition used in the WGBS analysis; "gene" and "TEs" in Fig.4e; "protein-coding genes" in Fig.4f. Is it the same as DE analysis (ie Cufflinks) which have been updated in the revision? Also, in Fig.4f, labels on x-axis of upper plots are missing.

In **Fig. 4e**, we classified DMR by the following rules:

1, If a DMR is overlapped (at least 1bp) with a TE (either ATXTEXXXXX or ATXGXXXXX annotated as "transposable element gene"), we would assign it as "TE".

2, If a DMR has no overlap with TE and it is overlapped with protein coding gene, we would assign it as "gene".

3, If a DMR has no overlap with TE or gene and it is overlapped with pseudogene, rRNA, miRNA, snRNA, snoRNA, etc., we would assign it as "other".

4, All the remaining DMRs that has no overlap with annotation will be assigned as "Intergenic".

For the generation **Fig 4f**, we used all the AT[1-5]GXXXXX whose annotation is "protein coding gene" as "Gene". And we used all the AT[1-5]TEXXXXX as "TE".

The annotation definition of protein coding genes and TEs in **Fig.4f** are the same as that in **Supplemental Data 2** (DE analysis).

We have added labels on x-axis of upper plots in **Fig.4f**.

• In Supplemental Table 3, please clarify what column C and D represent. Are they FPKM values?

The column C and D represent FPKM values. we have clarified it in **Supplemental Data 2** (old name: Supplemental Table 3).

• l.387 and Fig. 6f, the browser annotation and genome positions are not readable, due to low resolution. Please make them readable, or clarify the genome location and the annotations in the legend (rather than just "a representative region").

As suggested, we have now made all of them readable in **Fig. 6f** and **Supplementary Fig. 9**.
